# MTFR2 promotes VDAC1 oligomerization to reprogram BCAA metabolism in tumor cells to polarize TAMs in LUAD

**DOI:** 10.1038/s41419-026-08871-2

**Published:** 2026-05-20

**Authors:** Yutong Ge, Tao Yu, Shaokun Yu, Ao Sun, Shiyu Zhang, Meiling Zhang, Qian Wang, Jia Liu, Xuan Zhang, Lulin Zeng, Kaihua Lu

**Affiliations:** 1 Department of Oncology, The First Affiliated Hospital of Nanjing Medical University, Nanjing, China; 2 Department of Oncology Center, The Affiliated Suzhou Hospital of Nanjing Medical University, Suzhou Municipal Hospital, Suzhou, China; 3 Department of Respiratory and Critical Care Medicine, Nanjing First Hospital, Nanjing Medical University, Nanjing, China; 4 Department of Radiation Oncology, the First Affiliated Hospital of Nanjing Medical University, Nanjing, China

**Keywords:** Non-small-cell lung cancer, Cancer microenvironment, Cancer metabolism

## Abstract

Tumor-associated macrophages (TAMs) enriched in tumor microenvironment (TME) promote immune evasion and poor prognosis. Mitochondrial dysfunction, especially branched-chain amino acid (BCAA) metabolic reprogramming, has been confirmed to be involved in regulating TAMs function. The mitochondria-related protein MTFR2 was significantly upregulated in LUAD and closely associated with reduced survival. We conducted flow cytometry, multiplex immunofluorescence, immunohistochemistry on LUAD tissues, combined with analysis of public single-cell sequencing datasets, to characterize the TME features under MTFR2 dysregulation. Transcriptomics, metabolomics, and proteomics analyses revealed BCAA metabolic reprogramming, which was driven by MTFR2 binding to Leu125 within the β8 strand of VDAC1 and promoting its oligomerization. This interaction triggered mtDNA release into the cytoplasm, activating the TLR9-NF-κB pathway to upregulate BCAT1. Elevated concentrations of BCAA metabolites such as α-ketoisocaproate (KIC) and α-keto-β-methylvalerate (KMV) in the TME promoted M2 polarization and infiltration of TAMs by in vitro co-culture, 3D spheroid, and organoid models, and polarized M2 macrophages reciprocally promoted LUAD progression. Our findings establish a LUAD associated MTFR2-VDAC1-BCAT1 axis regulating the BCKAs-M2 axis in TAMs. Notably, targeting BCAT1 or depleting macrophages blocked this loop, offering a potential combination therapy for LUAD.

## Introduction

Lung cancer is a highly prevalent malignant tumor of the respiratory system worldwide, with the highest mortality rate globally [1, 2]. Approximately 48% of lung cancer patients present with distant metastases at diagnosis due to the lack of specific symptoms in the early stage, and these patients have a 5-year relative survival rate of 8% [3]. Lung adenocarcinoma (LUAD) is the most common histological subtype of non-small cell lung cancer. Its tumor microenvironment (TME) is particularly rich in immunosuppressive cells, such as tumor-associated macrophages (TAMs) [4]. In poorly differentiated solid and micropapillary adenocarcinomas, the proportion of macrophages reaches 13.21% and 15.71%, respectively [5]. TAMs, especially the M2 subtype with anti-inflammatory and pro-reparative phenotypes, contribute to tumor progression by secreting growth factors that promote tumor cell proliferation and angiogenesis, ultimately accelerating tumor malignancy [6, 7]. TAMs also suppress immune cell functions by secreting inhibitory cytokines such as IL-10 and TGF-β, expressing immune checkpoint molecules, depleting amino acids, and recruiting immunosuppressive cells [8–10]. Therefore, elucidating novel mechanisms underlying LUAD progression and immunosuppressive microenvironment formation is essential.

Tumor metabolic reprogramming critically regulates TAM polarization and infiltration [6]. Branched-chain amino acids (BCAAs) and their metabolites, branched-chain ketoacids (BCKAs), are pivotal modulators of the TME [11–15]. Recent studies have shown that BCKAs produced by tumor cells are taken up by macrophages, reducing their phagocytic activity [16]. However, the regulation of macrophage polarization by BCKAs such as ketoisocaproic acid (KIC), ketomethylvaleric acid (KMV), and ketoisovaleric acid (KIV) has not been fully elucidated [17, 18].

The crosstalk between mitochondrial dysfunction and metabolic reprogramming represents a cutting-edge area in tumor research [19–22]. Mitochondrial fission regulator 2 (MTFR2) is significantly overexpressed in multiple malignancies, correlating with poor prognosis [23–25]. MTFR2, a member of the MTFR1 family located on chromosome 6q23.3 and containing a conserved polyproline sequence, promotes mitochondrial fission [26]. In our previous study, we identified MTFR2 among mitochondria-related genes as a prognostic biomarker for LUAD [27]. MTFR2, a regulator of mitochondrial architecture and function, negatively correlates with immune cell infiltration in gastric cancer, implicating TME remodeling [25]. Peritumoral activated hepatic stellate cells promote hepatocellular carcinoma progression via MTFR2-mediated fatty acid oxidation [28]. However, the contribution of MTFR2 to amino acid metabolic reprogramming and its interactions with mitochondrial membrane proteins governing mitochondrial dysfunction and downstream signaling remain to be elucidated.

Voltage-dependent anion channel protein (VDAC1), a mitochondrial outer membrane protein regulating permeability, metabolite exchange, and mtDNA release [29, 30], is a potential interacting partner of MTFR2 in tumor-associated mitochondrial pathway modulation. Structurally, its channel gating is governed by the N-terminal α-helix [31], whereas its β-barrel, formed by 19 β-strands, drives oligomerization. Specific strands have distinct roles: β1, β2, and β19 mediate monomer interactions for dimerization, a process stabilized by β16 and β17. Moreover, β8 and β16 contribute to the assembly of higher-order oligomers [32]. Functionally, VDAC1 oligomerization is strongly associated with mitochondrial dysfunction and cancer progression.

Our study demonstrated that MTFR2 is highly expressed in LUAD and specifically binds to the Leu125 residue within the β8-strand of VDAC1, promoting VDAC1 oligomerization. This induces mtDNA release into the cytoplasm, activating the TLR9-NF-κB pathway to upregulate BCAT1 and trigger BCAA metabolic reprogramming. Elevated concentrations of BCAA metabolites such as KIC and KMV in the TME promoted M2 polarization and infiltration of TAMs, which drive LUAD progression. Targeting BCAT1 or macrophage depletion can effectively block this loop, offering a potential strategy for LUAD combination therapy.

## Results

### MTFR2 facilitates LUAD progression through inducing CD206^+^TAMs infiltration

Mitochondria, the powerhouses of eukaryotic cells, it is crucial to identify mitochondria-associated DEGs in LUAD and clarify their regulatory roles in tumor progression and the TME. In our previous study, we identified DEGs in LUAD from TCGA database and performed intersection analysis with mitochondria-related genes from the MitoCarta 3.0 database. Through univariate, LASSO, and multivariate Cox regression analyses, we screened mitochondrial fission regulator 2 (MTFR2) as a risk prediction target for LUAD among mitochondria-related genes [27]. However, the specific oncogenic mechanism of MTFR2 in LUAD remains to be further investigated.

To explore the clinical significance of MTFR2, tumor tissue microarrays (TMA) from 80 LUAD patients were analyzed. The immunohistochemical score (H-score) of MTFR2 in tumor tissues was significantly higher than that in adjacent normal tissues (Fig. S1A, B, *P* < 0.01). Patients with high MTFR2 expression showed a greater proportion of pathological stage IV (Supplementary Table 1, *P* = 0.044). Transcriptional-level validation was further confirmed using TCGA-LUAD (Fig. S1C, *P* < 0.0001). Consistently, MTFR2 was significantly overexpressed in LUAD cell lines (A549, H1975, H1299, and PC9) relative to the normal human lung epithelial cell line BEAS-2B (B2B) (Fig. S1D, E). Based on TMA, multivariate COX analysis indicated that lymph node metastasis, distant metastasis, and high MTFR2 expression were independent risk factors (Supplementary Table 2). LUAD patients with MTFR2 in the top 25% exhibited significantly shorter overall survival (OS) compared to those with MTFR2 in the bottom 25% (Fig. S1F, *P* = 0.022). The prognostic value of MTFR2 was verified in three independent cohorts (TCGA-LUAD, GSE30219, GSE31210) (Fig. S1G). This suggests that MTFR2 may serve as a potential prognostic marker for LUAD.

**Fig. 1:**
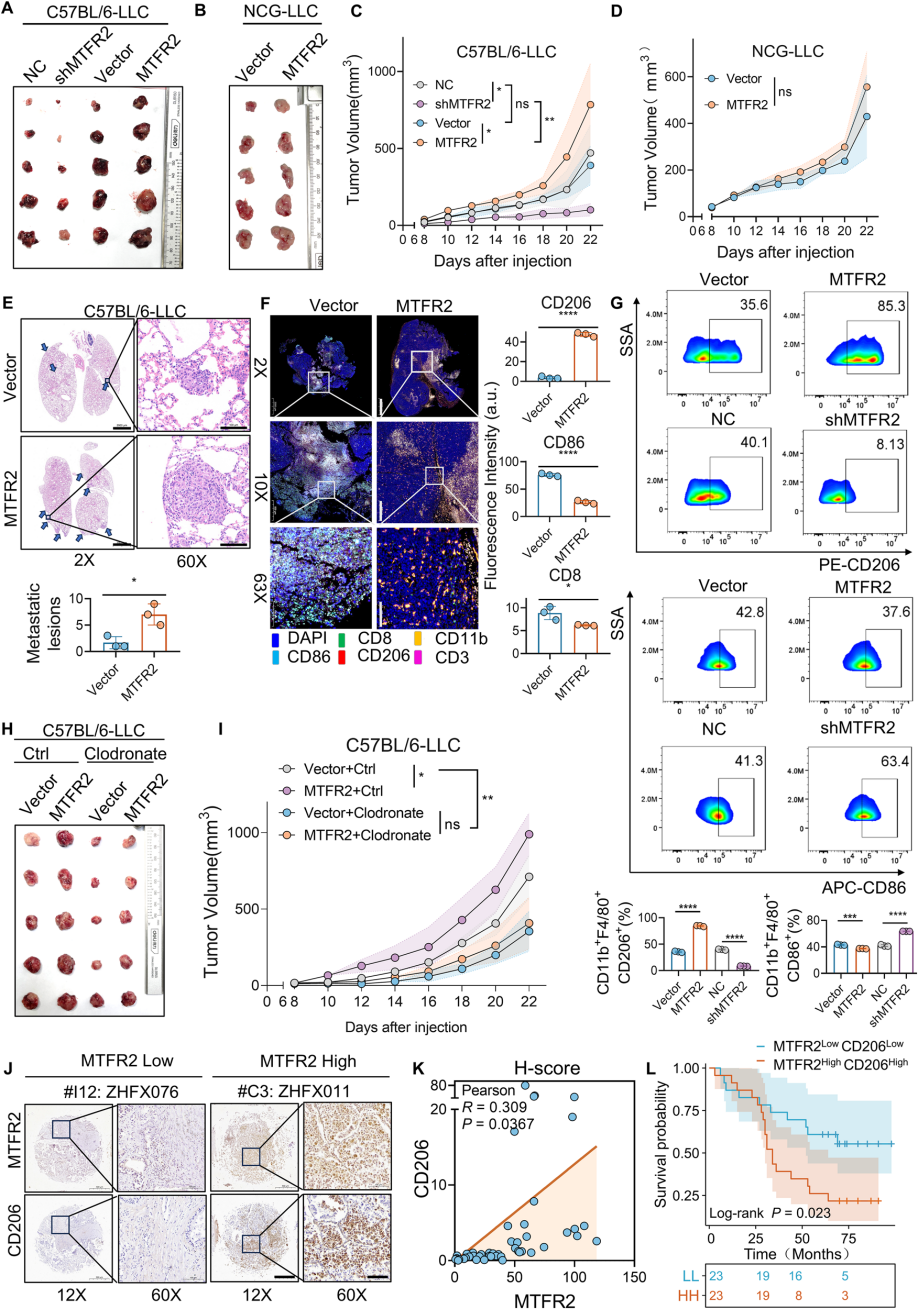
MTFR2 facilitates LUAD progression through inducing CD206^+^TAMs infiltration. Representative images of subcutaneous tumors in immunocompetent C57BL/6 **A** or immunodeficient NCG **B** mice axillary inoculated with 2 × 10^6^ stably transfected LLC-NC, LLC-shMTFR2, LLC-Vector, or LLC-MTFR2 cells (*n* = 5). Graphs depicting tumor volume over time for LLC-NC, LLC-shMTFR2, LLC-Vector, and LLC-MTFR2 groups in C57BL/6 **C** or NCG **D** mice (Data are represented as mean ± SD, *n* = 5, statistical analysis by two-way ANOVA). **E** Representative H&E staining images of whole lung tissues and quantification of lung metastatic lesions from C57BL/6 mice injected with 5 × 10^6^ stably transfected LLC-Vector or LLC-MTFR2 cells via the tail vein (Data are represented as mean ± SD, *n* = 3, statistical analysis by two-tailed Student’s *t*-test). **F** Representative multiplex immunofluorescence images of LLC-Vector and LLC-MTFR2 tumor sections in C57BL/6 mice, staining with antibodies against CD86 (cyan), CD8 (green), CD206 (red), CD11b (yellow), and CD3 (magenta) with DAPI nuclear counterstain (blue). Scale bars: 500 μm (10×) and 100 μm (63×). (Data are represented as mean ± SD, quantification based on ≥3 random fields per group, statistical analysis by two-tailed Student’s *t*-test). **G** Flow cytometric analysis showing the percentages of CD11b^+^F4/80⁺CD206^+^ and CD11b^+^F4/80⁺CD86⁺ macrophages in LLC-NC, LLC-shMTFR2, LLC-Vector, or LLC-MTFR2 groups of C57BL/6 mice (Data are represented as mean ± SD, *n* = 3, statistical analysis by one-way ANOVA). **H** Representative images of subcutaneous tumors in C57BL/6 mice pretreated with control liposomes or clodronate liposomes (1 mg/200 μL) after injection of 2 × 10^6^ LLC-Vector or LLC-MTFR2 cells (*n* = 5). **I** Graphs depicting tumor volume over time in C57BL/6 mice for LLC-Vector+Ctrl, LLC-MTFR2+Ctrl, LLC-Vector+Clodronate, and LLC-MTFR2+Clodronate groups (Data are represented as mean ± SD, *n* = 5, statistical analysis by two-way ANOVA). **J** Representative images of MTFR2 and CD206 IHC staining in the LUAD TMA. Scale bars: 500 μm (12×), 100 μm (60×). **K** Pearson correlation between MTFR2 and CD206 H-scores in the LUAD TMA. **L** Kaplan–Meier survival curves showing the OS difference between MTFR2^Low^CD206^Low^ and MTFR2^High^CD206^High^ groups based on the LUAD TMA. (ns not significant; *P < 0.05; **P < 0.01; ***P < 0.001; ****P < 0.0001).

We established xenograft models in immunocompetent C57BL/6 mice and immunodeficient NCG mice. In C57BL/6 mice, the MTFR2 overexpression group (LLC-MTFR2) exhibited a significantly increased tumor growth rate relative to the control group (LLC-Vector). By contrast, the knockdown group (LLC-shMTFR2) showed obvious growth inhibition (Fig. 1A, C, *P* < 0.05). Immunohistochemical (IHC) staining revealed that the Ki67 positive rate was significantly elevated in the LLC-MTFR2 group (Fig. S1H), while this group also exhibited a significantly higher final tumor weight than the LLC-Vector group (Fig. S1I, *P* < 0.05). In addition, MTFR2 overexpression significantly promoted lung metastasis formation (Fig. 1E, *P* < 0.05). Notably, no significant difference in tumor growth was observed between the LLC-MTFR2 and LLC-Vector groups in NCG immunodeficient mice (Fig. 1B, D and S1I).

Multiplex immunofluorescence (mIF) staining demonstrated that MTFR2 overexpression in vivo significantly increased CD206^+^ TAMs infiltration (*P* < 0.0001) but reduced CD86⁺ TAMs and CD8⁺ T cell infiltration compared with the LLC-Vector group (Fig. 1F). Flow cytometry analysis confirmed these results and showed that the proportion of CD11b⁺F4/80⁺ macrophages was elevated in LLC-MTFR2 tumors (Fig. S1J, *P* < 0.0001). Among these macrophages, the percentage of CD206^+^ TAMs was markedly increased while that of CD86⁺ TAMs was decreased (Fig. 1G). The percentage of CD11b⁺F4/80⁺CD206^+^ TAMs was correspondingly reduced in the LLC-shMTFR2 group (Figs. 1G and S1J). To delineate the role of TAMs in mediating MTFR2-indcued tumor promotion, macrophages in C57BL/6 mice were depleted using clodronate liposomes. Validation of macrophage depletion efficiency by flow cytometry and qPCR showing reduced CD11b⁺F4/80⁺ macrophages following clodronate administration (Fig. S1K, L). The difference in tumor growth between the LLC-Vector and LLC-MTFR2 group was completely abolished following macrophage depletion (Fig. 1H, I). Meanwhile, no significant differences were observed in the viability assays of 2D monolayers and 3D spheroids in vitro, as well as in the colony formation assays (Fig. S2A–C), further corroborating that MTFR2 indeed fulfills its biological function through modulation of the TME.

IHC validation on TMA (*n* = 80) showed significantly higher CD206 expression in MTFR2-high tumor tissues (Fig. 1J), and MTFR2 H-Score was positively correlation with CD206 H-Score (*R* = 0.309, *P* < 0.05, Fig. 1K). Patients with co-high expression of MTFR2 and CD206 had a significantly shorter median survival time than those with double-low expression (Fig. 1L and Supplementary Table 3). Taken together, our findings indicate that MTFR2 exerts its primary pro-tumor effects by regulating CD206⁺ TAMs to reshape the TME in vivo.

### MTFR2 recruits macrophages and promotes M2 polarization in vitro

**Fig. 2:**
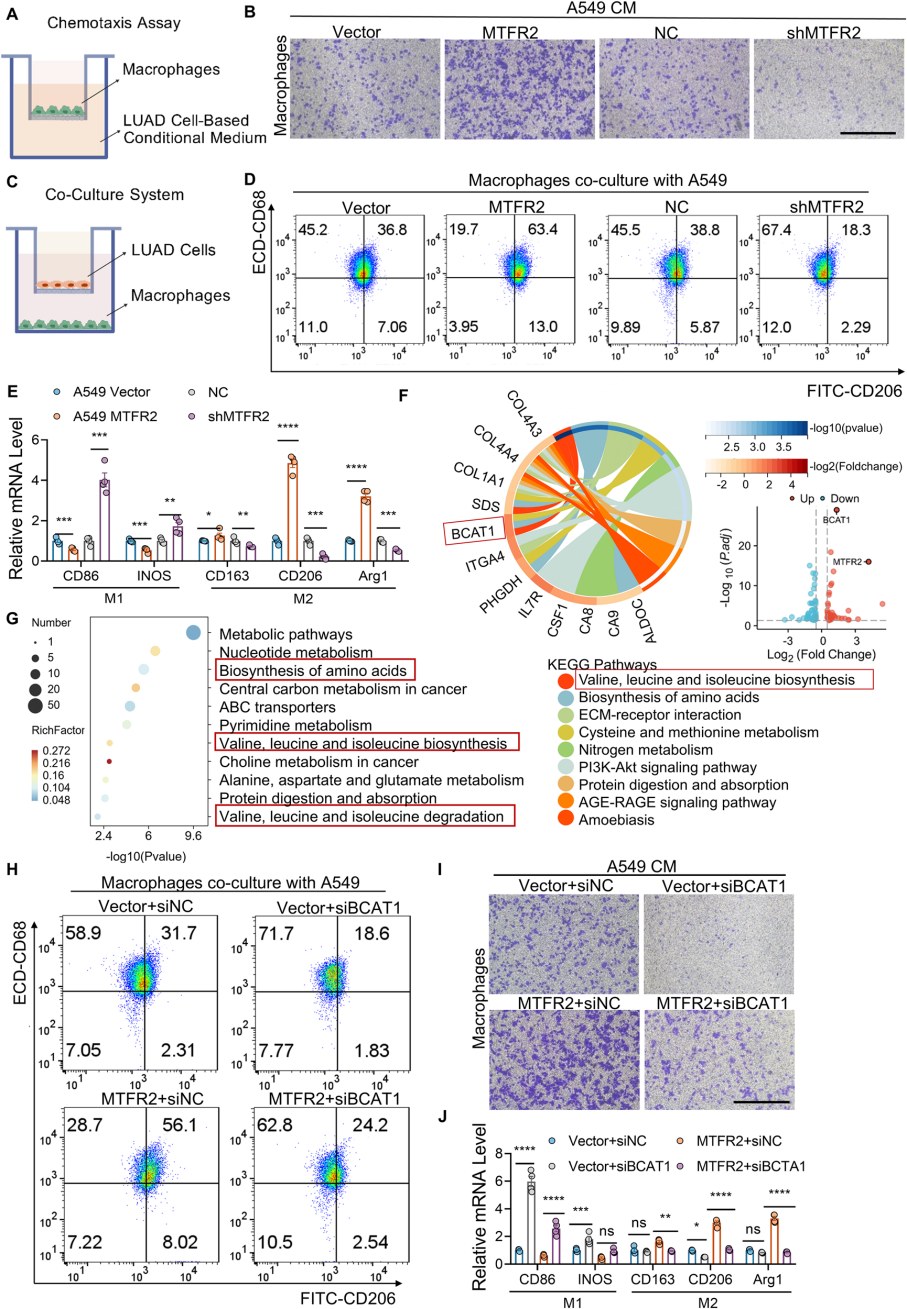
MTFR2 regulates chemotaxis and M2 polarization of macrophages via BCAT1. **A** Schematic illustration of the in vitro chemotaxis assay using THP-1-derived macrophages or RAW264.7 macrophages. **B** Transwell assay evaluating the effect of CM from A549-NC, A549-shMTFR2, A549-Vector, or A549-MTFR2 cells on the chemotaxis of THP-1-derived macrophages. **C** Schematic illustration of the co-culture system consists of THP-1-derived macrophages RAW264.7 macrophages, and LUAD cells under different conditions. **D** Flow cytometric analysis of the percentage of CD68⁺CD206^+^ cells in THP-1-derived macrophages after co-culture with A549-NC, A549-shMTFR2, A549-Vector, or A549-MTFR2 cells. **E** qPCR analysis of M1 (CD86, iNOS) or M2 (CD163, CD206, Arg1) markers in THP-1-derived macrophages after co-culture with A549-NC/A549-shMTFR2, or A549-Vector/A549-MTFR2 cells. Expression was normalized to ACTB as an internal control, with A549-NC and A549-Vector as references, respectively (Data are represented as mean ± SD, *n* = 3, analyzed by two-tailed Student’s *t*-test). **F** KEGG enrichment analysis of differentially expressed genes (DEGs) between A549-MTFR2 and A549-Vector cells. R software package clusterProfiler (version 3.14.3) was used to obtain the results of gene set enrichment (*n* = 3, minimum gene set size = 5, maximum gene set size = 5000, |log₂FC| > 1, adjusted *P* < 0.05). **G** Bubble plot of KEGG functional enrichment analysis for differential metabolites in the supernatant of A549-Vector and A549-MTFR2 cells (*n* = 3). **H** Flow cytometric analysis of the percentage of CD68⁺CD206^+^ cells in THP-1-derived macrophages after co-culture with A549 cells subjected to combined interventions (Vector+siNC, Vector+siBCAT1, MTFR2+siNC, or MTFR2+siBCAT1). **I** Transwell assay evaluating the effect of CM from A549 cells subjected to combined interventions (Vector+siNC, Vector+siBCAT1, MTFR2+siNC, or MTFR2+siBCAT1) on the chemotaxis of THP-1-derived macrophages. **J** qPCR analysis of M1 (CD86, iNOS) or M2 (CD163, CD206, Arg1) markers in THP-1-derived macrophages after co-culture with A549 cells subjected to combined interventions (Vector+siNC, Vector+siBCAT1, MTFR2+siNC, or MTFR2+siBCAT1). Expression was normalized to ACTB as an internal control, with Vector+siNC as the reference (Data are represented as mean ± SD, *n* = 3, analyzed by one-way ANOVA). (ns not significant; *P < 0.05; **P < 0.01; ***P < 0.001; ****P < 0.0001).

**Fig. 3:**
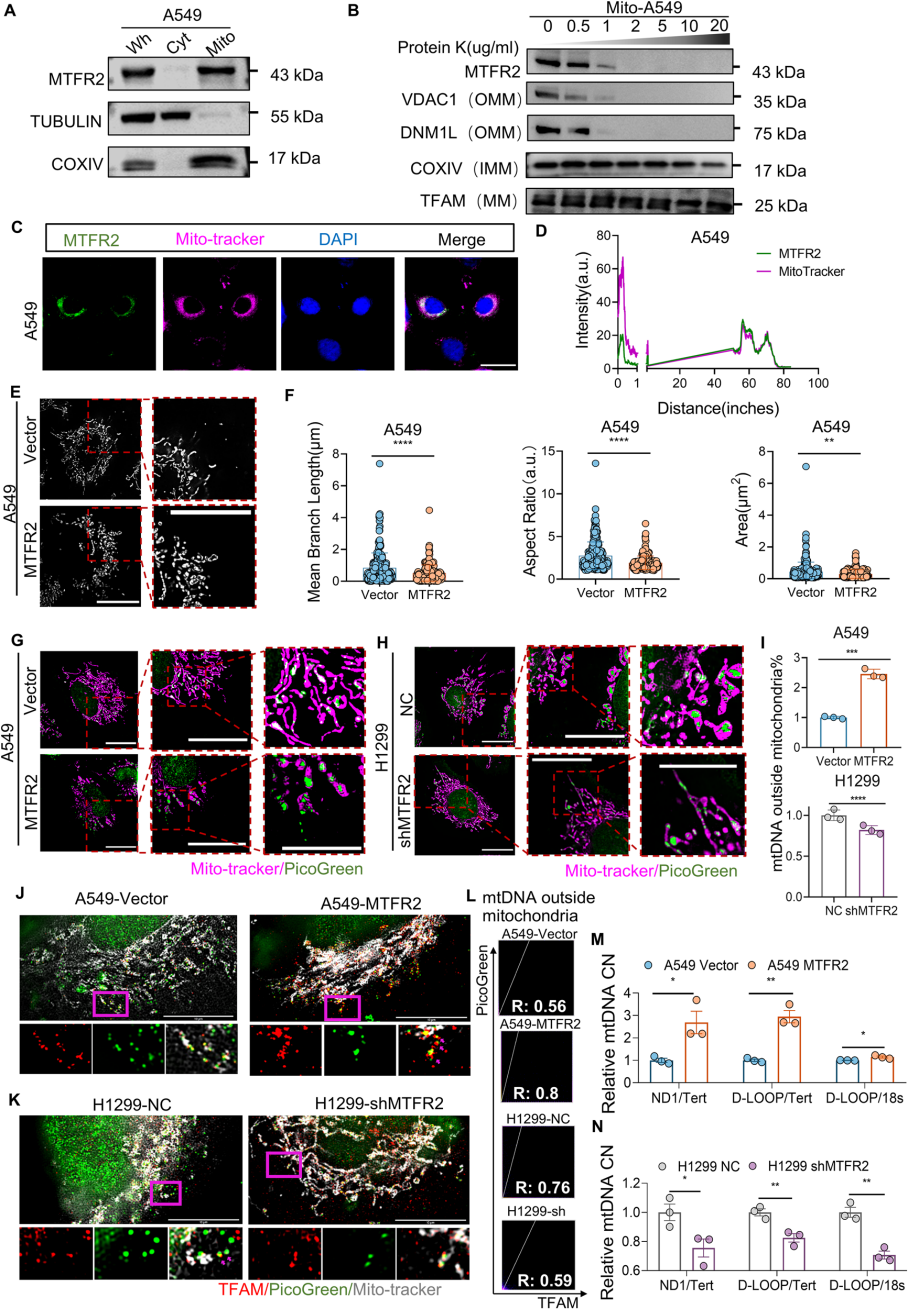
Induction of mtDNA release is a representative mitochondrial regulatory function of MTFR2. **A** Mitochondrial (Mito) and cytosolic (Cyt) proteins were extracted from A549 cells using the cell mitochondria isolation kit, with whole-cell lysates (Wh) extracted in parallel. Western blot was performed to detect the subcellular localization of MTFR2 in A549 cells. TUBULIN and COXIV served as cytosolic and mitochondrial protein markers, respectively. **B** Proteinase K protection assay to determine the localization of MTFR2 in mitochondrial membranes of A549 cells. Highly purified mitochondria from A549 cells were incubated with 0, 0.5, 1, 2, 5, 10, or 20 μg/ml proteinase K for 20 min, followed by Western blot analysis of MTFR2 localization. Outer mitochondrial membrane (OMM) proteins VDAC1 and DNM1L, inner mitochondrial membrane (IMM) protein COXIV, and mitochondrial matrix (MM) protein TFAM were used as markers for OMM, IMM, and MM, respectively. **C**, **D** Super-resolution confocal microscopy to detect co-localization of MTFR2 (green) and mitochondria (magenta, Mito-Tracker) in A549 cells; nuclei were counterstained with DAPI (blue). Scale bar, 50 μm; at least 3 random fields were selected per group (**C**). Fluorescence intensity and co-localization of MTFR2 and mitochondria in A549 cells were quantified using the Plot Profile plugin in ImageJ (D). **E** A549-Vector and A549-MTFR2 cells were incubated with 200 nM Mito-Tracker at 37 °C for 30 min, and mitochondrial network structure was observed using a multimodal super-resolution live cell microscope, with representative images shown. Scale bar, 20 μm; at least 3 random fields were selected per group. **F** Mitochondrial average branch length, average area, and aspect ratio in A549 cells were quantified using the Mitochondria Analyze plugin in ImageJ (Data are represented as mean ± SD, *n* = 3, analyzed by two-tailed Student’s *t*-test). **G–I** A549-Vector and A549-MTFR2 cells (**G**), or H1299-NC and H1299-shMTFR2 cells **H** were treated with 200 nM Mito-Tracker (magenta, 37 °C for 30 min) and PicoGreen (green, 37 °C for 1 h), followed by observation using a multimodal super-resolution live cell microscope, with representative images shown. Scale bar, 20 μm, 20 μm, 10 μm; at least 3 random fields were selected per group. dsDNA signals outside mitochondrial boundaries and excluding nuclear regions were quantified using ImageJ **I** (Data are represented as mean ± SD, *n* = 3, analyzed by two-tailed Student’s *t*-test). **J–L** Representative Multi-SIM super-resolution confocal microscopy images of A549-Vector and A549-MTFR2 cells (**J**), or H1299-NC and H1299-shMTFR2 cells (**K**) stained with Mito-Tracker (silver), PicoGreen (green), and CY3-TFAM (red). Scale bars, 10 μm. Magenta arrows indicate cytosolic mtDNA. At least 3 random fields were selected per group. Colocalization of PicoGreen and CY3-TFAM signals outside mitochondrial boundaries and excluding nuclear regions was quantified using the Coloc 2 plugin in ImageJ and subjected to Pearson correlation analysis (**L**). **M** Mitochondria-depleted cytosolic fractions of high purity were extracted from A549-Vector and A549-MTFR2 cells using a cell mitochondria isolation kit. DNA was extracted from cytosolic fractions and corresponding whole cells using a DNA extraction kit. qPCR was performed to amplify mitochondrial DNA (mtDNA) in cytosolic DNA using ND1 and D-LOOP primers, and nuclear DNA (nDNA) using TERT and 18 s primers. Results were normalized to TERT and 18 s, with A549-Vector as the reference (Data are represented as mean ± SD, *n* = 3, analyzed by two-tailed Student’s *t*-test). **N** Mitochondria-depleted cytosolic fractions of high purity were extracted from H1299-NC and H1299-shMTFR2 cells using a cell mitochondria isolation kit. DNA was extracted from cytosolic fractions and corresponding whole cells using a DNA extraction kit. qPCR was performed to amplify mtDNA in cytosolic DNA using ND1 and D-LOOP primers, and nDNA using TERT and 18 s primers. Results were normalized to TERT and 18 s, with H1299-NC as the reference (Data are represented as mean ± SD, *n* = 3, analyzed by two-tailed Student’s *t*-test). (ns not significant; *P < 0.05; **P < 0.01; ***P < 0.001; ****P < 0.0001).

To evaluate the recruitment ability of LUAD, THP-1-derived macrophages or RAW264.7 macrophages were seeded in the upper chamber, and tumor cell-conditioned medium (CM) was added to the lower chamber (Fig. 2A). Compared with the control groups (A549-NC, A549-Vector), the number of migrating macrophages was significantly lower in the group treated with CM from MTFR2-knockdown A549 (A549-shMTFR2) (*P* < 0.05), and higher in the group treated with CM from MTFR2-overexpressed A549 (A549-MTFR2) (Figs. 2B and S3J, *P* < 0.0001). Similarly, CM from H1299-shMTFR2 cells exhibited weaker macrophage recruitment ability (Fig. S3A, J, *P* < 0.0001). The number of migrating macrophages was increased in the MTFR2-overexpressed H23 cells (H23-MTFR2) group and LLC-MTFR2 group (Figs. 3B, C and S2J). These results suggest that MTFR2 can regulate macrophage chemotaxis.

We further examined the effect of MTFR2 on macrophage polarization using a separated co-culture system (Fig. 2C). LUAD cells were seeded in the upper chamber, and THP-1-derived macrophages or RAW264.7 macrophages were seeded in the lower chamber. The proportion of CD68⁺CD206^+^ macrophages (M2-type) was upregulated when co-cultured with A549-MTFR2, H23-MTFR2, or LLC-MTFR2 shown by Flow cytometry, but downregulated when co-cultured with A549-shMTFR2 or H1299-shMTFR2 (Figs. 2D and S3D–F, S3K). In addition, MTFR2 overexpression in tumor cells significantly upregulated mRNA expression of M2-type macrophage markers (CD163, CD206, Arg1) and downregulated that of M1-type markers (CD86, iNOS) in macrophages (Figs. 2E and S3G–I). Analysis of the GSE131907 single-cell dataset also revealed that MTFR2 is most highly expressed in epithelial cells, where its expression positively correlates with that of immunosuppressive macrophages (spp1⁺, areg⁺) and monocytes (vcan⁺) (Fig. S4A–C).

### MTFR2 regulates chemotaxis and M2 polarization of macrophages via BCAT1

To identify downstream effects of MTFR2 overexpression, RNA sequencing was performed. KEGG pathway analysis showed a significant enrichment in the BCAA metabolic pathway (Fig. 2F, log₂FC = 1.34, *P* < 0.0001). Concurrently, BCAT1, a key enzyme in BCAA metabolism, was significantly upregulated, and knockdown of MTFR2 could reverse the expression of BCAT1 (Fig. S5A–C). We further validated these findings in human clinical specimens. IHC analysis of a LUAD TMA cohort (*n* = 80) showed that MTFR2 expression was significantly elevated in BCAT1-high tumors (Fig. S5D), and MTFR2 H-Score was positively correlated with BCAT1 H-Score (*R* = 0.7026, *P* < 0.0001, Fig. S5E).

To verify whether MTFR2 regulates cell-autonomous BCAA metabolism, we cultured A549-Vector and A549-MTFR2 cells in complete medium for 24 h, then in BCAA-deficient medium for another 48 h. A Supernatants were collected for non-targeted metabolomics analysis, and KEGG enrichment analysis further showed differential metabolites enriched in the BCAA metabolic pathway (Figs. 2G and S5F). Therefore, we speculate that BCAT1 may be a key molecule through which MTFR2 regulates macrophage function. BCAT1 knockdown could reverse the polarization of CD68⁺CD206^+^ macrophages induced by MTFR2 overexpression (Figs. 2H and S5I, *P* < 0.0001). The ability of CM from MTFR2-overexpressed cells to recruit macrophages was also impaired by BCAT1 knockdown (Figs. 2I and S5J, *P* < 0.0001). MTFR2 overexpression-induced upregulation of M2 markers (CD163, CD206, Arg1) could be inhibited by BCAT1 knockdown, while M1 markers (CD86, iNOS) showed the opposite trend (Fig. 2J). Similar results were obtained using the specific BCAT1 inhibitor BCATc Inhibitor2 (BCATc Inh2) (Fig. S5G–K).

### Induction of mtDNA release is a representative mitochondrial regulatory function of MTFR2

To explore the regulatory mechanism of the MTFR2-BCAT1-M2 axis, the subcellular localization and functional characteristics of MTFR2 were examined. Mitochondria-cytoplasm fractionation assays showed that MTFR2 was mainly enriched in mitochondrial components, with its distribution consistent with that of the mitochondrial marker COXIV (Fig. 3A). With increasing proteinase K concentration, MTFR2 was degraded synchronously with outer mitochondrial membrane proteins VDAC1 and DNM1L, whereas inner mitochondrial membrane protein COXIV and mitochondrial matrix protein TFAM remained stable (Fig. 3B). This result confirmed that MTFR2 was specifically localized to the outer mitochondrial membrane. IF staining showed high overlap between MTFR2 and Mito-Tracker fluorescence signals, and co-localization analysis revealed consistent trends in their fluorescence intensities (Fig. 3C, D). The specific localization of MTFR2 to the outer mitochondrial membrane was further validated in H1299 cells (Fig. S6A–D), suggesting that MTFR2 may exert functions by regulating mitochondrial structure. Super-resolution microscopy revealed significant fragmentation of the mitochondrial network in A549-MTFR2 cells, characterized by reduced branch length and interrupted continuity (Fig. 3E). Mitochondrial morphological analysis showed that MTFR2 overexpression significantly shortened average mitochondrial branch length, reduced average area, and decreased aspect ratio (length/width) (Fig. 3F), whereas MTFR2 knockdown in H1299 cells significantly reversed these changes (Fig. S6E, F), indicating that MTFR2 participates in structural remodeling by promoting mitochondrial fission.

Abnormal mitochondrial structure is typically accompanied by functional alterations. Significantly reduced mitochondrial reactive oxygen species (ROS) level in A549-MTFR2 cells was observed, along with markedly decreased fluorescence intensity of Tetramethylrhodamine (TMRM), a marker of mitochondrial membrane potential (ΔΨm) (Fig. S6G–L, *P* < 0.0001), indicating MTFR2 overexpression impairs mitochondrial functional integrity. More importantly, PicoGreen staining combined with super-resolution imaging showed that obvious dsDNA signals appeared in the cytoplasm of A549-MTFR2 cells. In contrast, dsDNA in the control group was mainly confined to mitochondria (Fig. 3G, I). Conversely, knockdown of MTFR2 in H1299 cells resulted in dsDNA being more restricted to elongated mitochondria compared with the NC group (Fig. 3H, I). To exclude interference from nuclear DNA (nDNA), given that cytosolic dsDNA may comprise both mtDNA and nDNA, we performed immunofluorescence staining of TFAM in fixed cells and quantified the colocalization between TFAM and dsDNA to confirm the identity of cytosolic dsDNA as mtDNA, which showed a positive correlation outside mitochondrial contours (*R* ranging from 0.56 to 0.80, Fig. 3J, L). qPCR quantitative analysis further confirmed significantly increased cytoplasmic mtDNA levels (ND1 and D-LOOP) in A549-MTFR2 cells, which were significantly decreased in H1299-shMTFR2 cells (Fig. 3M, N). Above results suggest that MTFR2-induced cytoplasmic mtDNA release may be a key event for initiating downstream signals.

### MTFR2 engages VDAC1 Leu125 to facilitate oligomerization and elicit cytoplasmic mtDNA release

**Fig. 4:**
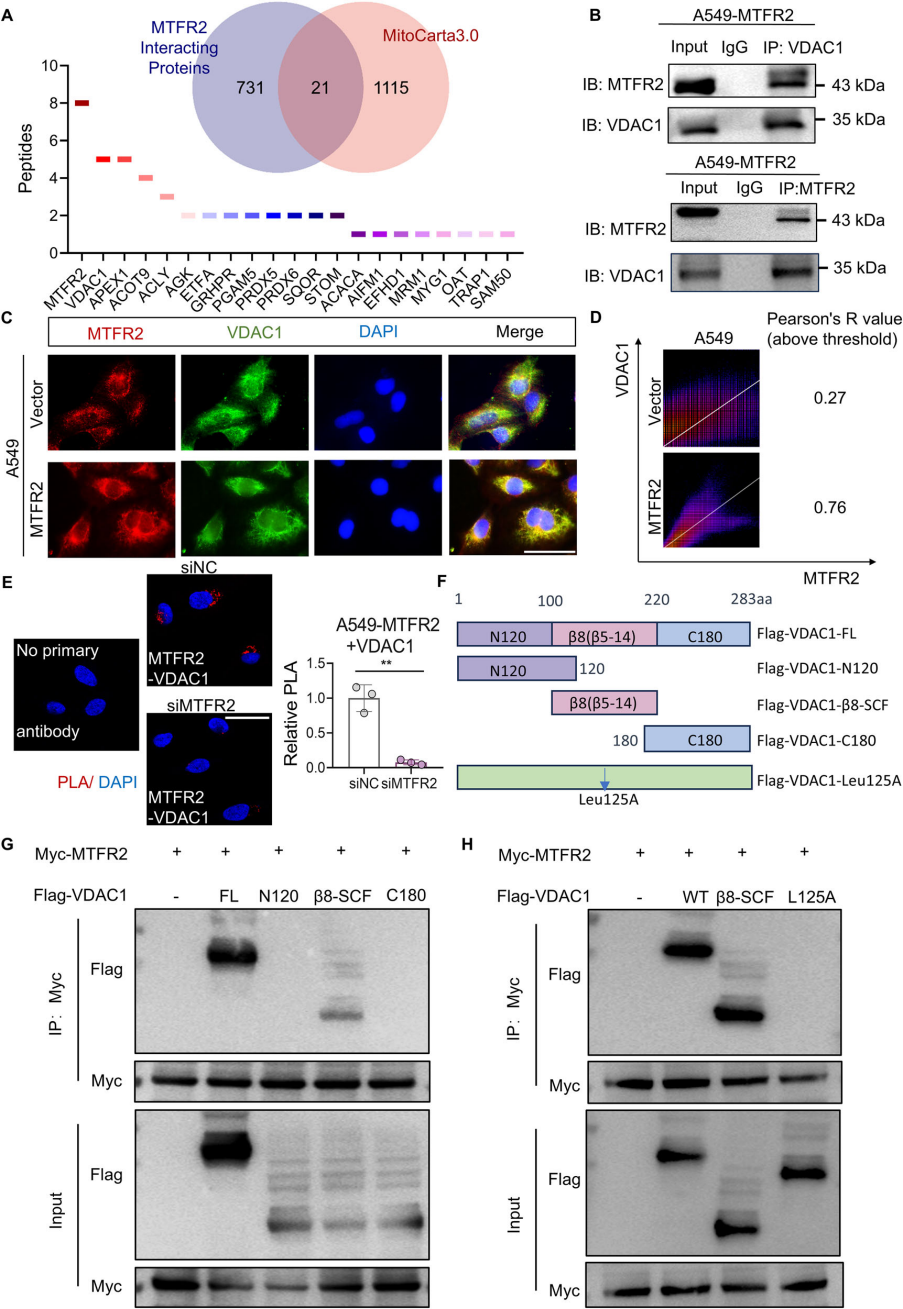
MTFR2 Engages VDAC1 Leu125. **A** Mass spectrometry identified 752 MTFR2-binding proteins, among which 21 are specifically localized to mitochondria. These 21 mitochondrial proteins interacting with MTFR2 are ranked by the number of enriched peptides in descending order. **B** Co-immunoprecipitation assay to verify the interaction between VDAC1 and MTFR2. **C**, **D** Confocal microscopy to detect co-localization of MTFR2 (red) and VDAC1 (green) in A549-Vector and A549-MTFR2 cells. Nuclei were visualized by DAPI (blue). Scale bar, 50 μm, at least 3 random fields were selected per group (**C**). Fluorescence intensity and co-localization of MTFR2 and VDAC1 in A549-Vector and A549-MTFR2 cells were quantified using the Coloc 2 plugin in ImageJ (**D**). **E** After overexpressing MTFR2 and VDAC1 in A549 cells, the cells were treated with siMTFR2 and siNC, respectively, and the interaction between MTFR2 and VDAC1 was detected by PLA (red). Nuclei were visualized by DAPI (blue). Scale bar, 30 μm, at least 3 random fields were selected per group. Fluorescence intensity was quantified using ImageJ (Data are represented as mean ± SD, *n* = 3, analyzed by two-tailed Student’s *t*-test). **F** Schematic diagram of full-length (FL) VDAC1 and its truncation mutants, as well as the point mutant. Three VDAC1 truncation mutants were designed: N-terminal truncation (N120, 1–120 aa), β8-strand-containing fragment (β8-SCF, 100–220 aa, encompassing β5–14 strands), and C-terminal truncation (C180, 180–283 aa). The Leu125Ala (L125A) point mutant was generated by substituting leucine at position 125 with alanine. All constructs were Flag-tagged. **G** Co-IP assay verifying the interaction between MTFR2 and VDAC1 truncation mutants. Flag-tagged VDAC1-FL, N120, β8-SCF, or C180 was co-transfected into HEK-293T cells with Myc-tagged MTFR2. Cell lysates were immunoprecipitated with anti- Myc antibody, followed by immunoblotting with anti-Myc and anti-Flag antibodies. Input lysates were probed to confirm protein expression. **H** Co-IP assay confirming the indispensability of Leu125 for VDAC1-MTFR2 interaction. Flag-tagged VDAC1-WT, β8-SCF, or L125A was co-transfected into HEK-293T cells with Myc-tagged MTFR2. Co-IP and immunoblotting were performed as in (**G**) (ns, not significant; *P < 0.05; **P < 0.01; ***P < 0.001; ****P < 0.0001).

Coomassie brilliant blue staining revealed specific protein bands in MTFR2 immunoprecipitation (IP) products (Fig. S7A). Subsequent mass spectrometry identified 752 potential interacting proteins, 21 of which were mitochondrial-localized (Supplementary Table 4). Notably, the outer mitochondrial membrane protein VDAC1 was the most prominent candidate interacting protein based on peptide match counts (Fig. 4A). Co-IP experiments further confirmed the specific binding between MTFR2 and VDAC1 (Fig. 4B). IF staining revealed colocalization of MTFR2 and VDAC1 in A549 cells (*R* = 0.27). MTFR2 overexpression significantly enhanced their colocalization, with a marked increase in the Pearson’s correlation coefficient (Fig. 4C, D, *R* = 0.76). This interaction pattern was independently validated in H1299-NC and H1299-shMTFR2 cells (Fig. S7B, C). Furthermore, after overexpressing MTFR2 and VDAC1 in A549 cells, Proximity ligation assay (PLA) detection showed that the interaction between MTFR2 and VDAC1 was significantly reduced following siMTFR2 treatment (Fig. 4E).

Based on structural prediction using AlphaFold3 and visualization analysis with PyMOL, the potential binding site of MTFR2 on VDAC1 was identified as Leu125 (Fig. S7D), which localizes to the β8-strand domain of VDAC1. To validate this prediction and delineate the critical domain mediating their interaction, three sets of VDAC1 truncation mutants were designed: an N-terminal truncation (1–120 aa, N120), a β8- strand-containing-fragment (100–220 aa, encompassing β5–14 strands, β8-SCF), and a C-terminal truncation (180–283 aa, C180) (Fig. 4F). Flag-tagged expression vectors for full-length (FL) VDAC1 and each truncation mutant were constructed, and co-transfected into HEK-293T cells together with Myc-tagged MTFR2. Co-IP assay demonstrated that only VDAC1-FL and β8-SCF exhibited substantial binding to MTFR2, whereas no interaction was detected with the VDAC1-N120 or C180 truncations (Fig. 4G). These findings indicate that the β8-strand domain of VDAC1 serves as the core structural motif mediating its interaction with MTFR2.

To further confirm the indispensability of the Leu125 residue, a Flag-tagged VDAC1 Leu125Ala (L125A) mutant was generated (Fig. 4F). Co-transfection of Myc-MTFR2 with either wild-type VDAC1 (WT), β8-SCF, or L125A mutant into HEK-293T cells followed by Co-IP assays revealed that the L125 A mutation completely abrogated the binding between VDAC1 and MTFR2, in contrast to the strong interaction observed with WT and β8-SCF (Fig. 4H). PLA further validated that VDAC1-WT and VDAC1-β8-SCF did not affect the interaction between VDAC1 and MTFR2. In contrast, the red fluorescent signal was significantly reduced in the VDAC1-L125A mutant group, indicating a marked attenuation of their interaction (Fig. S7E). Collectively, these results establish that the interaction between MTFR2 and VDAC1 is highly specific, and that both the β8-strand domain of VDAC1 and its key residue Leu125 are indispensable for this interaction. Additionally, levels of both MTFR2 and BCAT1 were significantly positively correlated with VDAC1 in TCGA-LUAD (Fig. S7F, G), indicating a synergistic expression pattern of the three in LUAD.

**Fig. 5:**
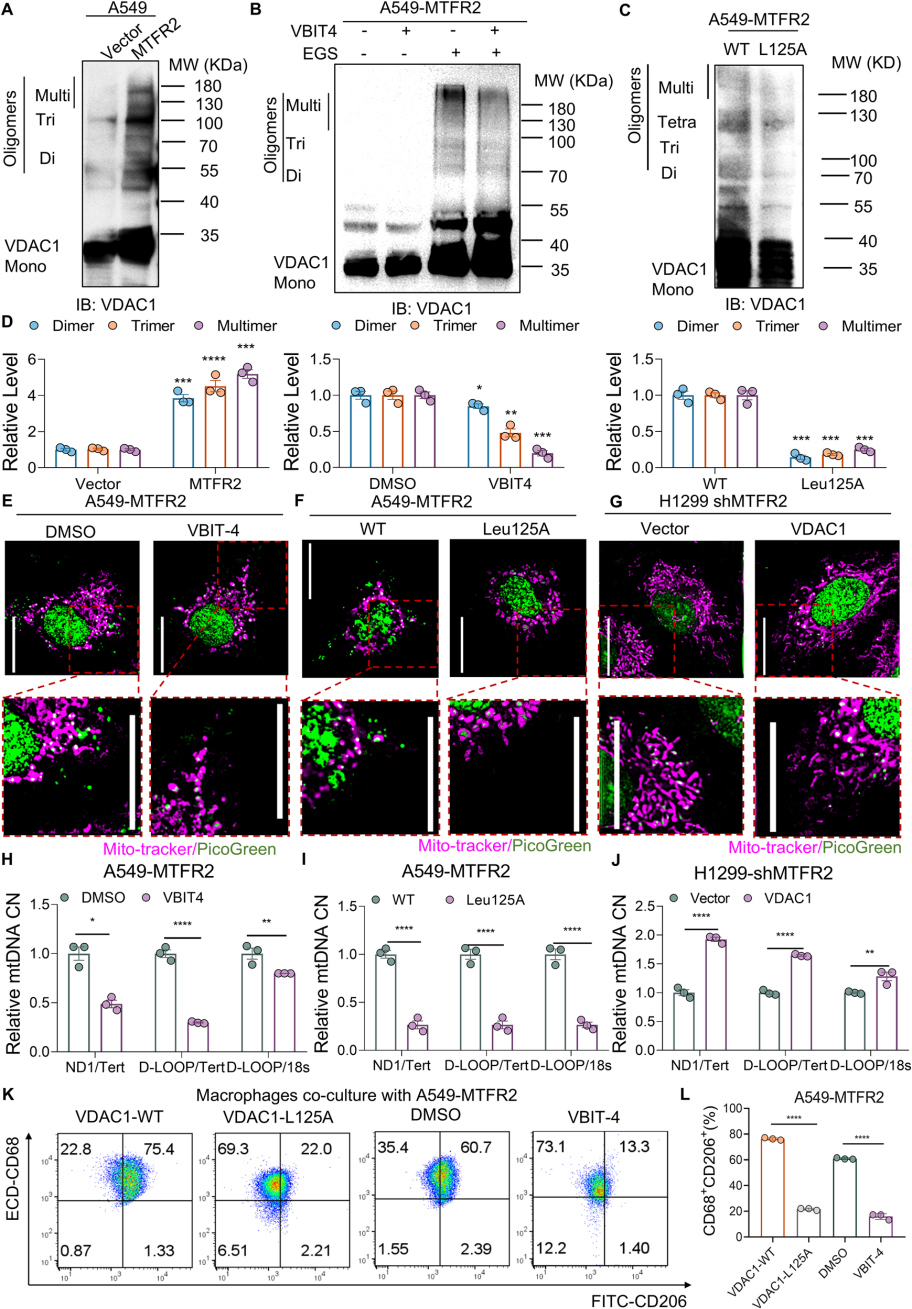
MTFR2 engages VDAC1 Leu125 to facilitate oligomerization and elicit cytoplasmic mtDNA release. **A** Western blot analysis of VDAC1 oligomerization in A549-Vector and A549-MTFR2 cells. The cross-linking agent EGS was used to stabilize oligomer structures during electrophoresis. **B** Western blot analysis of VDAC1 oligomerization in A549-MTFR2 cells with or without pretreatment with the VDAC1 oligomerization inhibitor VBIT4 (10 μM). The cross-linking agent EGS was used to stabilize oligomer structures during electrophoresis. **C** Western blot analysis of VDAC1 oligomerization in A549-MTFR2 cells with or without transfection of the VDAC1 L125A mutant. The cross-linking agent EGS was used to stabilize oligomer structures during electrophoresis. **D** Quantification results of oligomerization in **A**–**C** (Data are represented as mean ± SD, *n* = 3, analyzed by two-tailed Student’s t-test). (**E**) A549-MTFR2 cells treated with VBIT4 (VDAC1 oligomerization inhibitor) (10 μM) or DMSO were stained with 200 nM Mito-Tracker (incubated at 37 °C for 30 min) and PicoGreen (incubated at 37 °C for 1 h), followed by observation using multimodal super-resolution live cell microscope, with representative images shown. Scale bar, 20 μm; at least 3 random fields were selected per group. **F** A549-MTFR2 cells with or without transfection of the VDAC1 L125A mutant were stained with 200 nM Mito-Tracker (incubated at 37 °C for 30 min) and PicoGreen (incubated at 37 °C for 1 h), followed by observation using multimodal super-resolution live cell microscope, with representative images shown. Scale bar, 20 μm; at least 3 random fields were selected per group. **G** H1299-shMTFR2 cells transfected with VDAC1 overexpression plasmid or empty vector were stained with 200 nM Mito-Tracker (incubated at 37 °C for 30 min) and PicoGreen (incubated at 37 °C for 1 h), followed by observation using multimodal super-resolution live cell microscope, with representative images shown. Scale bar, 20 μm; at least 3 random fields were selected per group. **H** Mitochondria-depleted cytosolic fractions of high purity were extracted from A549-MTFR2 cells treated with VBIT4 (10 μM) or DMSO using a cell mitochondria isolation kit. DNA was extracted from cytosolic fractions and corresponding whole cells using a DNA extraction kit. qPCR was performed to amplify mitochondrial DNA (mtDNA) in cytosolic DNA using ND1 and D-LOOP primers, and nuclear DNA (nDNA) using TERT and 18 s primers. Results were normalized to TERT and 18 s, with A549-MTFR2 + DMSO as the reference (Data are represented as mean ± SD, *n* = 3, analyzed by two-tailed Student’s *t*-test). **I** Mitochondria-depleted cytosolic fractions of high purity were extracted from A549-MTFR2 cells with or without transfection of the VDAC1 L125A mutant using a cell mitochondria isolation kit. DNA was extracted from cytosolic fractions and corresponding whole cells using a DNA extraction kit. qPCR was performed to amplify mitochondrial DNA (mtDNA) in cytosolic DNA using ND1 and D-LOOP primers, and nuclear DNA (nDNA) using TERT and 18 s primers. Results were normalized to TERT and 18 s, with A549-MTFR2 WT as the reference (Data are represented as mean ± SD, *n* = 3, analyzed by two-tailed Student’s *t*-test). **J** Mitochondria-depleted cytosolic fractions of high purity were extracted from H1299-shMTFR2 cells transfected with VDAC1 overexpression plasmid or empty vector using a cell mitochondria isolation kit. DNA was extracted from cytosolic fractions and corresponding whole cells using a DNA extraction kit. qPCR was performed to amplify mtDNA in cytosolic DNA using ND1 and D-LOOP primers, and nDNA using TERT and 18 s primers. Results were normalized to TERT and 18 s, with H1299-shMTFR2 as the reference (Data are represented as mean ± SD, *n* = 3, analyzed by two-tailed Student’s *t*-test). **K**, **L** Flow cytometric analysis of the percentage of CD68⁺CD206⁺ cells in THP-1-derived macrophages after co-culture with A549-MTFR2 cells subjected to different interventions (VDAC1-WT and VDAC1-L125A, or VBIT4 (10 μM) and DMSO) (Data are represented as mean ± SD, *n* = 3, analyzed by two-tailed Student’s *t*-test). (ns not significant; *P < 0.05; **P < 0.01; ***P < 0.001; ****P < 0.0001).

Oligomerization of VDAC1 is a key event that mediates mtDNA release, a process driven by specific regions on its β-barrel structure, such as the β2-β5 and β11-β14 strands [32]. Given that the interaction between MTFR2 and VDAC1 depends on the Leu125 residue within the β8-strand, we were prompted to investigate its impact on oligomerization. Western blot showed that the level of VDAC1 oligomers in A549-MTFR2 cells was significantly increased (Fig. 5A, D). Using VBIT4, the specific inhibitor of VDAC1 oligomerization, could effectively block MTFR2-induced VDAC1 oligomerization (Fig. 5B, D). Consistently, transfection of the VDAC1 L125A mutant, which disrupts the MTFR2-VDAC1 interaction, also completely blocked MTFR2-mediated oligomerization of VDAC1 (Fig. 5C, D). Super-resolution microscopy showed that VBIT4 treatment and transfection of the VDAC1 L125A mutant reduced free cytoplasmic mtDNA signals and restored the mitochondrial localization of mtDNA in A549-MTFR2 (Figs. 5E, F and S7H). qPCR quantification confirmed that VBIT4 treatment and transfection of the VDAC1 L125A mutant significantly reduced cytoplasmic mtDNA levels compared with the control group in A549-MTFR2 (Fig. 5H, I). In H1299 cells, VDAC1 overexpression reversed the inhibition of mtDNA release caused by MTFR2 knockdown (Figs. 5G, J and S7H). These results indicate that MTFR2 specifically enhances cytoplasmic mtDNA release by engaging to VDAC1 Leu125 and facilitating its oligomerization.

Pretreatment with VBIT4 or transfection of the VDAC1 L125A mutant reduced the proportion of CD68⁺CD206^+^ macrophages induced by A549-MTFR2 (Fig. 5K, L, *P* < 0.0001), and impaired macrophage chemotaxis (Fig. S7J, K, *P* < 0.0001). qPCR showed that the above treatments reversed M2 markers upregulation and restored M1 markers expression (Fig. S7I). Together, these results further demonstrate that MTFR2 binds to VDAC1 and promotes its oligomerization, and this process is critical for the ability of MTFR2 to drive M2 polarization.

### Cytoplasmic mtDNA upregulates BCAT1 via TLR9-NF-κB pathway to drive macrophage M2 polarization and chemotaxis

**Fig. 6:**
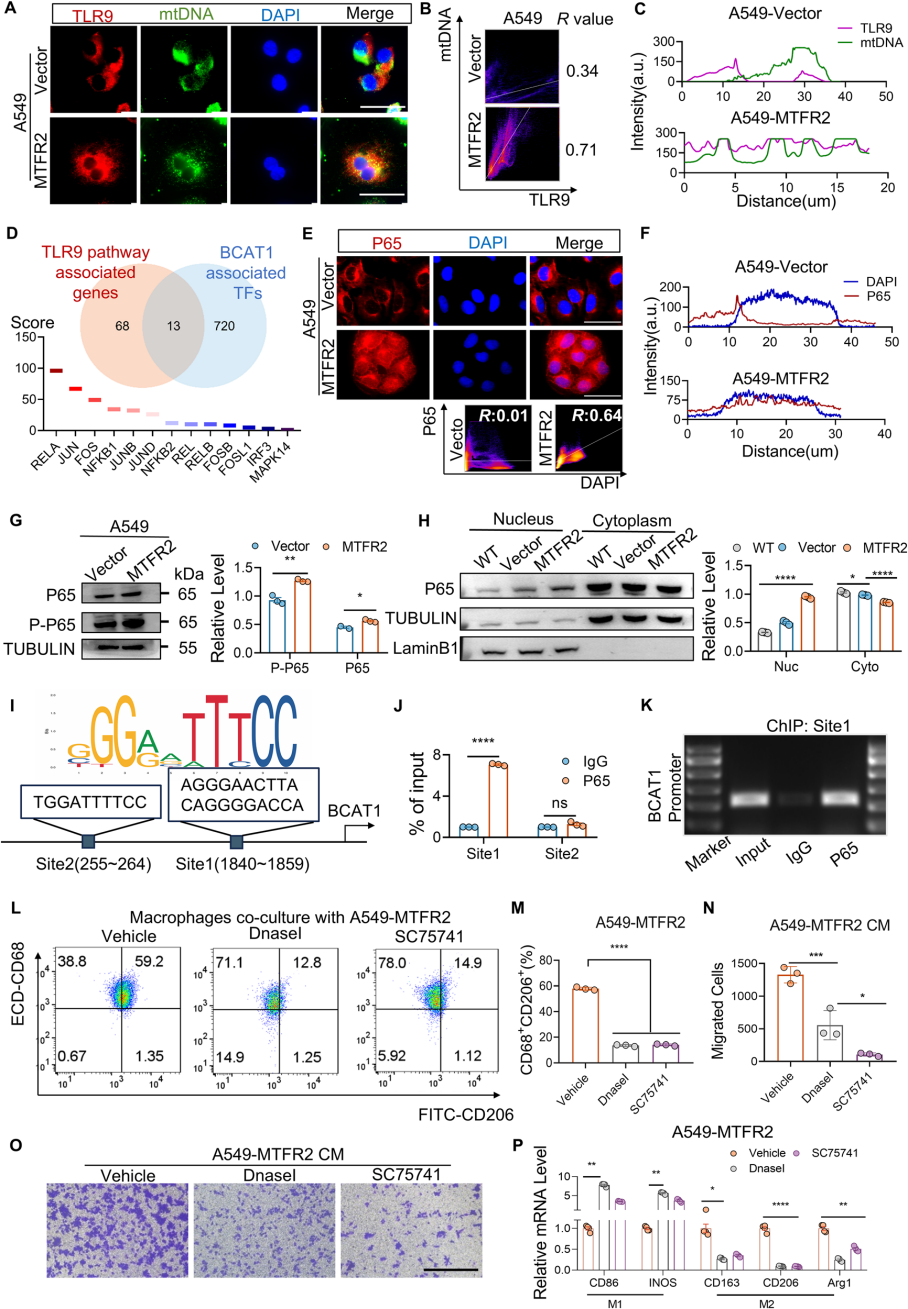
Cytoplasmic mtDNA drives macrophage M2 polarization and chemotaxis by upregulating BCAT1 via the TLR9-NF-κB pathway. **A–C** Confocal fluorescence microscopy to detect co-localization of TLR9 (red) and mtDNA (green) in A549-Vector and A549-MTFR2 cells. Nuclei were visualized by DAPI (blue). Scale bar, 50 μm; at least 3 random fields were selected per group (**A**). Pearson’s correlation coefficients for TLR9 and mtDNA co-localization were determined using the Coloc 2 plugin in ImageJ (**B**). Fluorescence intensity profiles along the indicated lines were generated using the Plot Profile plugin (**C**). **D** Venn diagram of TLR9 pathway genes and BCAT1 transcription factor sets, ranked by transcription factor prediction scores. **E**, **F** Confocal fluorescence microscopy to detect P65 (red) localization in A549-Vector and A549-MTFR2 cells. Nuclei were visualized by DAPI (blue). Scale bar, 50 μm; at least 3 random fields were selected per group. Pearson’s correlation coefficients for P65 and DAPI co-localization were determined using the Coloc 2 plugin in ImageJ (**E**). Fluorescence intensity profiles along the indicated lines were generated using the Plot Profile plugin (**F**). **G** Western blot analysis of P65 and phosphorylated P65 (P-P65) protein expression in A549-Vector and A549-MTFR2 cells, normalized to TUBULIN as an internal control (Data are represented as mean ± SD, *n* = 3, analyzed by two-tailed Student’s *t*-test). **H** Nuclear and cytosolic proteins were extracted from A549-WT, A549-Vector, and A549-MTFR2 cells using a nuclear-cytosolic isolation kit. Western blot was performed to detect the subcellular localization of P65. TUBULIN and LaminB1 served as cytosolic and nuclear protein markers, respectively (Data are represented as mean ± SD, *n* = 3, analyzed by one-way ANOVA). **I** Schematic diagram of 2 predicted RELA (P65) binding sites in the BCAT1 promoter region. ChIP assay to detect the interaction between P65 and BCAT1 promoter binding sites in A549-MTFR2 cells: qPCR quantitative analysis (**J**) and agarose gel electrophoresis validation (**K**). **L–M** Flow cytometric analysis of the percentage of CD68⁺CD206⁺^+^ cells in THP-1-derived macrophages after co-culture with A549-MTFR2 cells subjected to different interventions (DNase I, NF-κB inhibitor SC75741 (0.2 μM), or vehicle) (Data are represented as mean ± SD, *n* = 3, analyzed by one-way ANOVA). **N**, **O** Transwell assay to evaluate the effect of CM from A549-MTFR2 cells treated with DNase I, SC75741 (0.2 μM), or vehicle on the chemotactic of THP-1-derived macrophages (Data are represented as mean ± SD, *n* = 3, analyzed by one-way ANOVA). **P** qPCR analysis of M1 (CD86, iNOS) or M2 (CD163, ^+^, Arg1) markers in THP-1-derived macrophages after co-culture with A549-MTFR2 cells treated with DNase I, SC75741 (0.2 μM), or vehicle. Expression was normalized to ACTB as an internal control, with A549-MTFR2+ Vehicle as the reference (Data are represented as mean ± SD, *n* = 3, analyzed by one-way ANOVA). (ns not significant; *P < 0.05; **P < 0.01; ***P < 0.001; ****P < 0.0001).

As a typical damage-associated molecular pattern (DAMP), mtDNA released into the cytoplasm can be recognized by pattern recognition receptors (PRRs). RNA sequencing-based Reactome analysis and GSEA analysis indicate that the Toll-like receptor pathway was enriched in the MTFR2 overexpression group (Fig. S8A, B). Since TLR9 specifically recognizes CpG-rich mtDNA, we first detected their interaction. Confocal microscopy showed significantly stronger TLR9-mtDNA colocalization signals in A549-MTFR2 cells (*R* = 0.71) than in A549-Vector cells (*R* = 0.34) (Fig. 6A–C), suggesting that TLR9 is the main recognition receptor. To investigate whether other cytosolic DNA sensors contribute to mtDNA recognition in this context, we further examined the colocalization of mtDNA with cGAS and NLRP3 in A549-MTFR2 cells. Notably, while TLR9 exhibited strong colocalization with mtDNA (*R* = 0.71), both cGAS and NLRP3 showed minimal colocalization with mtDNA in the same cellular context (cGAS: *R* = 0.14; NLRP3: *R* = -0.08) (Fig. S8C–H). These results indicate that, in A549-MTFR2 cells, mtDNA preferentially associates with TLR9 rather than cGAS or NLRP3, supporting that TLR9 serves as the predominant receptor for mtDNA recognition upon MTFR2 overexpression. Intersection analysis of TLR9 pathway related genes and BCAT1 associated transcription factors (Supplementary Table 5) identified RELA (P65) as a key node mediating BCAT1 transcription (Fig. 6D). GSEA analysis also showed the enrichment of the NF-κB pathway in A549-MTFR2 (Fig. S8B, I). IF staining and nucleocytoplasmic fractionation experiments confirmed significantly increased P65 nuclear translocation and phosphorylated P65 (P-P65) protein expression in A549-MTFR2 cells (Fig. 6E–H), indicating MTFR2 overexpression activates the NF-κB signaling pathway. Using the VDAC1 inhibitor VBIT4 or clearing cytoplasmic mtDNA with DnaseI both significantly reduced P65 nuclear translocation (Fig. S8J–L), confirming cytoplasmic mtDNA is a key upstream signal for NF-κB activation.

BCAT1 promoter prediction identified two potential P65 binding sites (Fig. 6I). ChIP confirms that P65 directly bound to BCAT1 promoter site 1 in A549-MTFR2 cells (Fig. 6J, K, *P* < 0.0001). Pretreatment with DnaseI or NF-κB inhibitor SC75741 inhibited BCAT1 expression (Fig. S8M, *P* < 0.0001), reduced the proportion of CD68⁺CD206^+^ macrophages induced by A549-MTFR2 (Fig. 6L, M, *P* < 0.0001), and impaired macrophage chemotaxis (Fig. 6N, O, *P* < 0.0001). qPCR showed that the above treatments reversed M2 markers upregulation and restored M1 markers expression (Fig. 6P). Clinical analysis revealed that patients in the VDAC1 or TLR9 high-expression group exhibiting poor prognosis (Fig. S8N). In summary, cytoplasmic mtDNA promotes P65-mediated BCAT1 transcription in LUAD by activating TLR9, laying a molecular foundation for increased BCAT1 expression. However, as a key enzyme in BCAA metabolism, how BCAT1 generates specific products through catalytic metabolic reactions after upregulation, and subsequently acts directly on macrophages to regulate their polarization and chemotactic phenotypes, remains a key mechanism to be further explored.

### BCAT1 orchestrates BCAA metabolism to polarize TAMs toward M2 via KIC and KMV

**Fig. 7:**
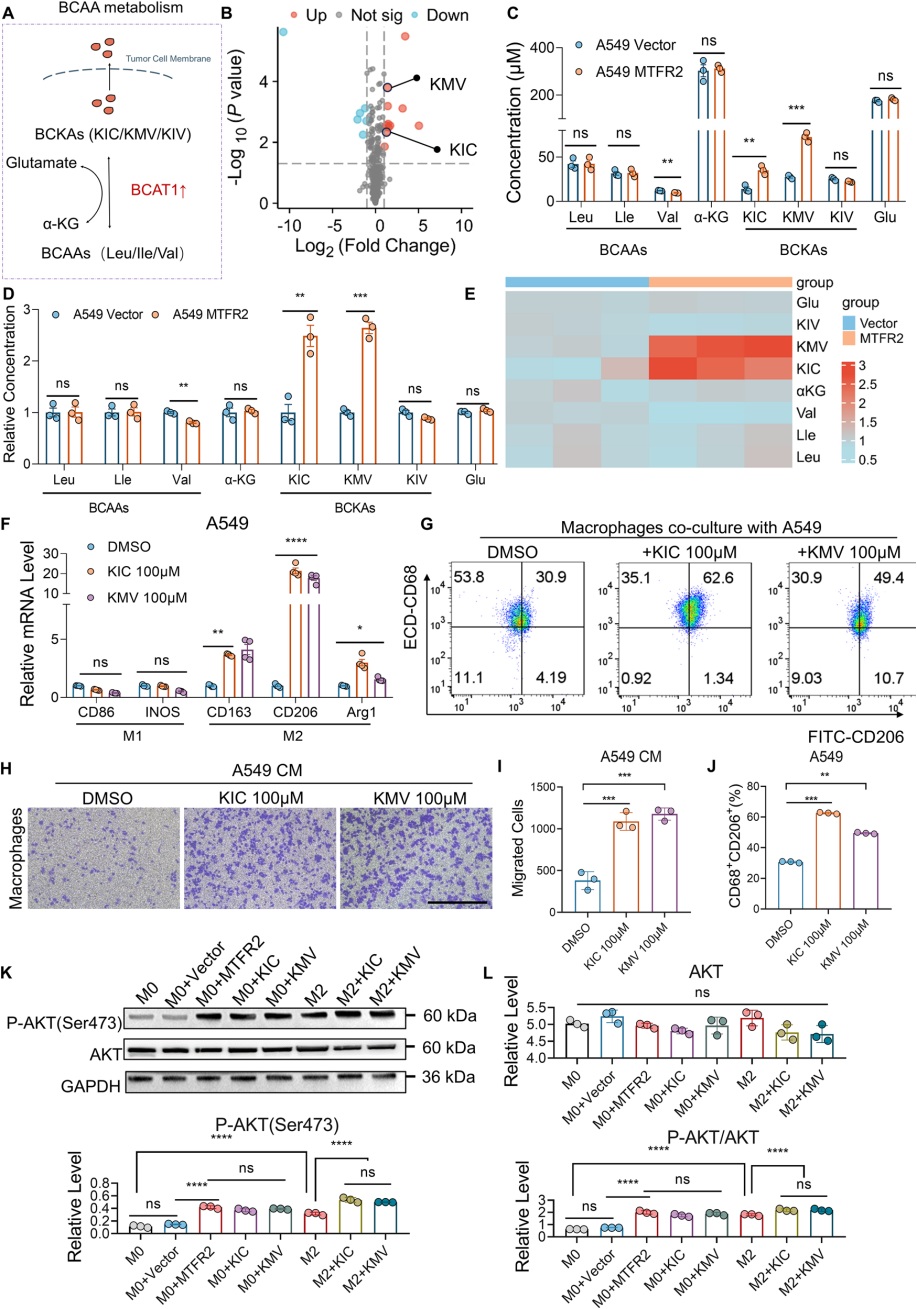
BCAT1-mediated BCAA metabolites KIC and KMV promote TAMs M2 polarization through mTORC2-AKT pathway. **A** Schematic illustration of the BCAA metabolic pathway mediated by BCAT1. **B** Volcano plot of differential metabolites in the supernatant of A549-Vector and A549-MTFR2 cells (*n* = 3). (**C**) Statistical analysis of the content of major metabolites in the BCAA metabolic pathway in the supernatant of A549-Vector and A549-MTFR2 cells (Data are represented as mean ± SD, *n* = 3, analyzed by two-tailed Student’s *t*-test). Statistical analysis of the relative content of major metabolites in the BCAA metabolic pathway in the supernatant of A549-Vector and A549-MTFR2 cells, normalized to A549-Vector as the reference (**D**), and corresponding heatmap (**E**) (Data are represented as mean ± SD, *n* = 3, analyzed by two-tailed Student’s *t*-test). **F** qPCR analysis of M1 (CD86, iNOS) or M2 (CD163, CD206, Arg1) markers in THP-1-derived macrophages after co-culture with A549 + DMSO, A549 + KIC (100 μM), or A549 + KMV (100 μM) cells. Expression was normalized to ACTB as an internal control, with A549 + DMSO as the reference (Data are represented as mean ± SD, n = 3, analyzed by one-way ANOVA). **G**, **J** Flow cytometric analysis of the percentage of CD68⁺CD206^+^ cells in THP-1-derived macrophages after co-culture with A549 + DMSO, A549 + KIC (100 μM), or A549 + KMV (100 μM) cells (Data are represented as mean ± SD, *n* = 3, analyzed by one-way ANOVA). **H**, **I** Transwell assay to assess the effect of conditioned medium from A549 + DMSO, A549 + KIC (100 μM), or A549 + KMV (100 μM) cells on the chemotactic capacity of THP-1-derived macrophages (Data are represented as mean ± SD, *n* = 3, analyzed by one-way ANOVA). **K**, **L** Total proteins were extracted from THP-1-derived macrophages treated under specified conditions (co-cultured with A549-Vector or A549-MTFR2, exogenous KIC/KMV supplementation, or exogenous KIC/KMV supplementation after IL-4-induced M2 polarization). Western blot was performed to detect the expression levels of AKT and phosphorylated AKT (at Ser473) (**A**). Quantification was normalized to TUBULIN as an internal control (Data are represented as mean ± SD, *n* = 3, analyzed by one-way ANOVA) (**L**). (ns, not significant; *P < 0.05; **P < 0.01; ***P < 0.001; ****P < 0.0001).

BCAT1 primarily catalyzes BCAA metabolic conversion (Fig. 7A). It reversibly converts BCAAs to BCKAs while transferring amino groups to α-KG to generate glutamic acid. BCKAs include KIC, KMV, and KIV, corresponding to metabolites of leucine, isoleucine, and valine, respectively. Metabolomic analysis to clarify BCAA metabolic reprogramming showed significantly increased KIC and KMV levels in A549-MTFR2 cell supernatants (Figs. 7B–E and S9). Initially, exogenous gradient supplementation of KIC and KMV was performed in M0 macrophages to identify the optimal concentrations (Fig. S10A, B). Then, exogenous KIC and KMV (100 μM) supplementation induced CD206 expression and migration in macrophages (Fig. 7F–J), an effect similar to MTFR2 overexpression. Western blot showed significantly increased phosphorylated AKT (Ser473), a specific substrate of mTORC2, in THP-1-derived macrophages co-cultured with A549-MTFR2. Exogenous addition of KIC and KMV could also achieve similar effects. In IL-4 induced M2 macrophages, KIC and KMV treatment further enhanced AKT phosphorylation (Fig. 7K, L), indicating that KIC and KMV can specifically activate the mTORC2-AKT pathway in macrophages. To determine whether KIC and KMV regulate macrophages polarization through AKT activation, we performed rescue experiments using the AKT phosphorylation inhibitor MK-2206. M0 macrophages treated with KIC or KMV (100 μM) or IL-4-induced M2 macrophages were exposed to increasing concentrations of MK-2206 (0, 1, 5, and 10 μM). Flow cytometry analysis demonstrated that MK-2206 dose-dependently inhibited the KIC/KMV-induced upregulation of CD206, indicating that AKT signaling plays a critical role in KIC/KMV-mediated M2 macrophage polarization (Fig. S10C–E).

**Fig. 8:**
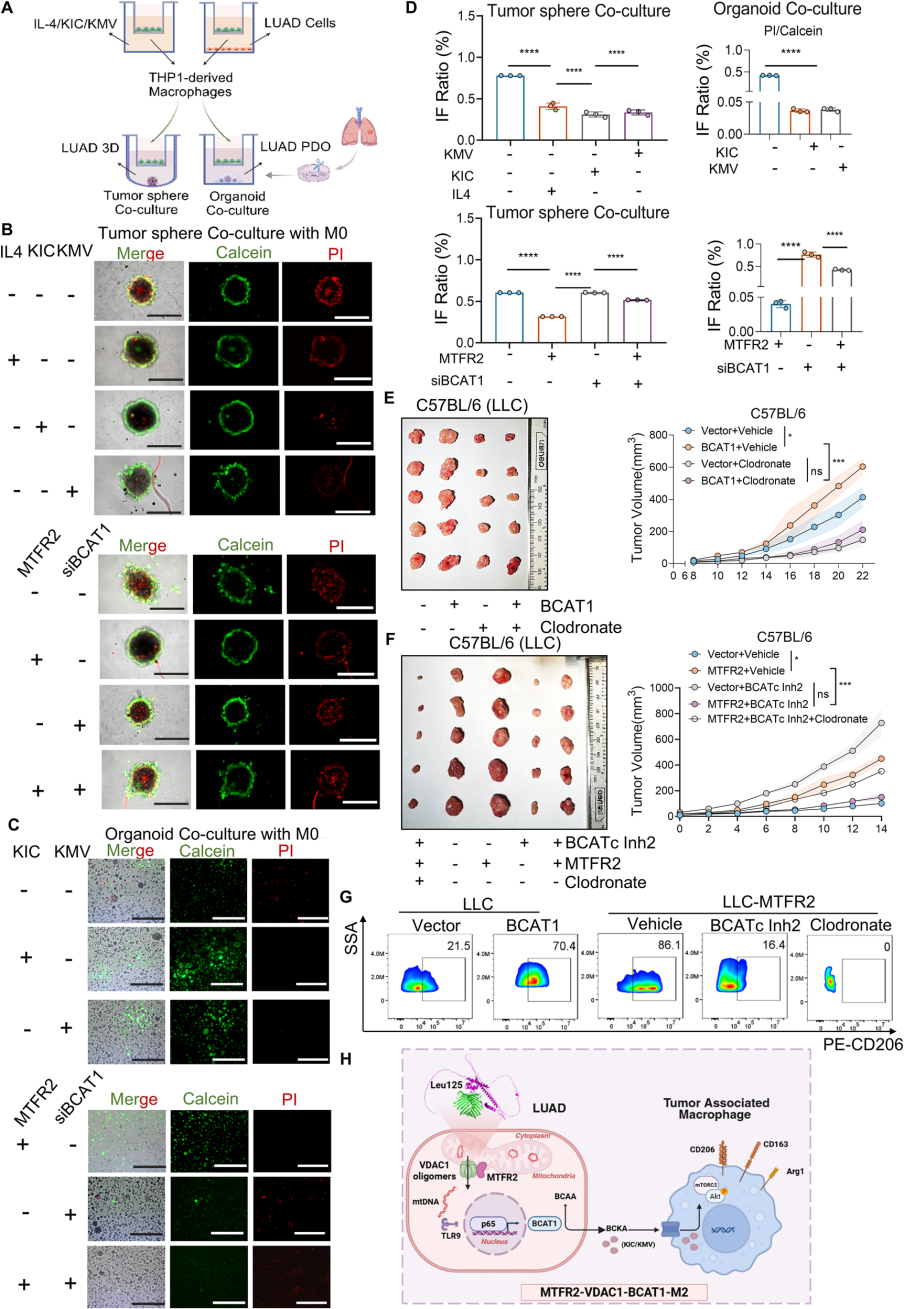
Targeting BCAT1 or depleting macrophages inhibits M2 polarization and tumor-promoting effects mediated by KIC and KMV. **A** Schematic diagram of tumor sphere- macrophage co-culture and organoid-macrophage co-culture. **B** THP1-derived macrophages pretreated with IL4, KIC, or KMV, or co-culture with A549-MTFR2 (with or without BCAT1 knockdown), were further co-cultured with A549 tumor spheres. At 96 h, spheres were incubated with Beyo3D™ 1X Calcein-AM and 1X PI dye at 37 °C for 10 min, followed by fluorescence intensity observation using laser confocal microscopy. Scale bar, 50 μm; at least 3 random fields were selected per group. **C** THP1-derived macrophages pretreated with KIC or KMV, or co-culture with A549-MTFR2 (with or without BCAT1 knockdown). The macrophages after the above co-culture treatments were further co-cultured with LUAD-PDO. After 48 h, samples were incubated with Beyo3D™ 1X Calcein-AM and 1X PI dye at 37 °C for 10 min, followed by fluorescence intensity observation using laser confocal microscopy. Scale bar, 50 μm; at least 3 random fields were selected per group. **D** Quantitative Analysis of PI/Calcein Fluorescence Intensity Ratio in **B**, **C** (Data are represented as mean ± SD, *n* = 3, analyzed by one-way ANOVA). **E** Graphs depicting tumor volume over time in C57BL/6 mice for LLC-Vector, LLC-BCAT1, LLC-Vector+Clodronate, and LLC- BCAT1+Clodronate groups (Data are represented as mean ± SD, *n* = 5, statistical analysis by two-way ANOVA). Representative images of subcutaneous tumors in C57BL/6 mice pretreated with control liposomes or clodronate liposomes (1 mg/200 μL) after injection of 2 × 10^6^ LLC-Vector or LLC-BCAT1 cells. Statistical analysis of tumor volume over 14 days (Data are represented as mean ± SD, *n* = 5, analyzed by two-way ANOVA with Tukey’s test). **F** C57BL/6 immunocompetent mice were pretreated with tail-vein clodronate liposomes (1 mg/200 μL) every 3 days starting 3 days prior to subcutaneous inoculation of 2 × 10⁶ LLC-Vector or LLC-MTFR2 cells in the axilla. Upon reaching ~3 mm tumor diameter, mice were administered BCATc Inh2 (30 mg/kg, i.p.) every 2 days for 14 days, either alone or in combination with continued clodronate treatment. Tumor growth was monitored for 14 days following treatment initiation (Data are represented as mean ± SD, *n* = 5, analyzed by two-way ANOVA with Tukey’s test). **G** Flow cytometric analysis of M2 macrophage infiltration in subcutaneous tumors from C57BL/6 mice. Percentage of CD11b⁺F4/80⁺CD206^+^ cells in LLC-Vector and LLC-BCAT1 groups (Left). Percentage of CD11b⁺F4/80⁺CD206⁺ macrophages in LLC-MTFR2-bearing mice treated with vehicle, BCATc Inh2 (30 mg/kg), or BCATc Inh2 plus clodronate liposomes (1 mg/200 μL) (Right). **H** Schematic diagram illustrating the mechanism of MTFR2 in regulating the interaction between LUAD and TAMs. (ns not significant; *P < 0.05; **P < 0.01; ***P < 0.001; ****P < 0.0001).

Using the co-culture model illustrated in Fig. 8A, we first subjected THP1-derived macrophages to pretreatment or co-culture treatments. These treatments included IL4 pretreatment, exogenous addition of KIC and KMV, or co-culture with A549-MTFR2 cells. Subsequently, the macrophages were co-cultured with 3D tumor spheroids. Results indicated that all the aforementioned treatments enhanced the viability of tumor spheroids. However, knockdown of BCAT1 in A549-MTFR2 cells abrogated this pro-tumorigenic effect of macrophages following co-culture with A549-MTFR2 cells (Fig. 8B, D). Furthermore, we established an organoid-macrophage co-culture model using PDOs from LUAD patients, which yielded consistent results as described above (Fig. 8C, D). To establish the functional necessity of AKT signaling in these observations, we pretreated macrophages with the selective AKT allosteric inhibitor MK-2206 (1, 5, and 10 μM) prior to co-culture with A549 spheroids. Pharmacological blockade of AKT dose-dependently abrogated the pro-tumorigenic effects of both IL-4-induced M2 macrophages and KIC/KMV-activated macrophages on tumor spheroid viability, whereas MK-2206 alone did not affect basal spheroid growth (Fig. S11A–D). These results demonstrate that AKT activation is indispensable for KIC/KMV-mediated macrophage-dependent tumor promotion.

In vivo experiments demonstrated that C57BL/6 mice subcutaneously inoculated with LLC-BCAT1 cells exhibited significantly faster tumor growth compared to the LLC-Vector group. In contrast, macrophage depletion eliminated this growth difference (Fig. 8E). Flow cytometry analysis revealed that infiltration of CD11b⁺F4/80⁺ macrophages was increased, among which the proportion of CD206^+^ TAMs was elevated while that of CD86⁺ TAMs was reduced, in subcutaneous LLC-BCAT1 tumors relative to the LLC-Vector group (Figs. 8G and S11E–G). Furthermore, C57BL/6 mice subcutaneously inoculated with LLC-MTFR2 cells also showed notably accelerated tumor growth compared to the LLC-Vector group. Intraperitoneal injection of BCATc Inh2 significantly slowed the growth of LLC-MTFR2 tumors, and combined pretreatment with tail-vein Clodronate further reduced tumor size (Fig. 8F). Flow cytometry analysis revealed that intraperitoneal administration of BCATc Inh2 diminished overall CD11b⁺F4/80⁺ macrophage infiltration and reversed the polarization imbalance, exhibiting decreased M2 and restored M1 proportions within this population, in LLC-MTFR2 tumors relative to vehicle-treated controls. In the macrophage depletion group, efficient ablation of total CD11b⁺F4/80⁺ macrophages was confirmed (>96% reduction), with both M1 and M2 subsets being concomitantly eliminated (Figs. 8G and S11E–G).

Our study demonstrates that MTFR2 specifically binds to the Leu125 residue within the β8-strand of VDAC1 and promotes VDAC1 oligomerization formation. This event subsequently induces the release of mtDNA into the cytoplasm, activating the TLR9-NF-κB pathway to upregulate BCAT1 and thereby triggering BCAA metabolic reprogramming. BCAA metabolites KIC and KMV activate mTORC2-AKT signaling in macrophages, driving M2 polarization. These M2 macrophages in turn promote LUAD progression. Notably, targeting BCAT1 or macrophage depletion can effectively block this loop, offering a potential strategy for LUAD combination therapy (Fig. 8H).

## Discussion

This study systematically investigates LUAD-TAM interaction, focusing on the role of BCAA reprogramming mediated by MTFR2 in TAM regulation. MTFR2 promotes VDAC1 oligomerization through specific binding to Leu125 of the VDAC1 β8-strand, inducing cytoplasmic mtDNA release, which activates the TLR9-NF-κB signaling, leading to BCAT1 upregulation. BCKAs catalyzed by BCAT1, particularly KIC and KMV, are internalized by TAMs to induce M2 polarization. M2-TAMs accelerate LUAD proliferation, forming a positive feedback loop for immune evasion. These findings identify MTFR2 as a core hub linking mitochondrial dysfunction to immunosuppressive TME, providing a theoretical basis for reversing LUAD immune evasion.

MTFR2 is highly expressed in multiple malignancies and strongly associated with poor prognosis [33–35]. Previous studies linked its pro-tumor mechanisms to Akt signaling activation or metabolic reprogramming, including fatty acid oxidation and glycolysis [27, 28, 36, 37]. We observed the high MTFR2 expression in LUAD, which correlates with reduced survival. However, unlike its role in other cancers, MTFR2 does not significantly affect autonomous LUAD cell proliferation. The GSE131907 single-cell dataset shows that MTFR2 expression positively correlates with immunosuppressive macrophages (spp1⁺, areg⁺) and monocytes (vcan⁺), suggesting involvement in TAMs regulation. Subcutaneous tumor models in immunocompetent C57BL/6 and severely immunodeficient NCG mice confirmed MTFR2 promotes tumor development by regulating the LUAD TME. Using TMA analysis, macrophage-depleted mouse models, and in vitro coculture systems, we demonstrated MTFR2 recruits TAMs and promotes M2 polarization, demonstrating distinct immunomodulatory activity. M2-TAMs correlate with poor prognosis across multiple malignancies, including breast, renal, gastric, and lung cancers. Tumor cells recruit macrophages into the tumor stroma and polarize of TAMs toward the M2 phenotype via cytokine and exosome release. Reciprocally, M2-TAMs facilitate tumor cell proliferation, drive metastasis through extracellular matrix remodeling, and promote angiogenesis by secreting growth factors, cytokines, and metalloproteinases [38]. Given the crucial regulatory role of M2-TAMs, MTFR2 may indirectly regulate LUAD proliferation by modulating macrophage chemotaxis and polarization.

We characterized MTFR2 localization, interactome, and function to clarify its role in M2 polarization. MTFR2 localizes to the outer mitochondrial membrane to promote fission, reduce ROS, decrease ΔΨm, and release mtDNA. We confirmed the mitochondrial origin of this cytosolic dsDNA through TFAM colocalization analysis, effectively excluding nuclear DNA contamination. As an endogenous DAMP, cytosolic or extracellular mtDNA triggers innate immune pattern recognition receptor activation and downstream signaling [39–42]. Cytoplasmic mtDNA release is regulated by excessive mitochondrial fragmentation, mitochondrial channel protein oligomerization, and ROS accumulation [43–46]. We found MTFR2 specifically interacts with outer mitochondrial membrane VDAC1 via the β8-domain, critically dependent on Leu125 within this strand. Substitution of Leu125 with Ala abolishes this interaction.

VDAC1 is a highly conserved channel protein whose distinct functional domains regulate oligomerization and mtDNA release. The N-terminal domain (residues 1–20) typically folds into an α-helix that regulates voltage-gating. Through conformational changes, it alters channel conductance states, modulating transport of ions and small-molecule metabolites between mitochondria and cytosol while also participating in VDAC1 oligomerization [31]. The central β-barrel domain (β1–β19 strands) forms the core channel structure and serves as the key interface for protein interactions and intermonomer contacts during oligomerization. The β8-strand integrity is required for VDAC1 transition to tetramers or higher-order oligomers [32]. The C-terminal domain ensures proper β-barrel folding and maintains VDAC1 localization as an outer membrane channel protein [47]. VDAC1 oligomerization represents a rate-limiting step in mtDNA release. Monomeric VDAC1 forms a narrow channel that restricts mtDNA passage, whereas oligomeric VDAC1 assembles into large-conductance pores in the outer membrane, enabling mtDNA transport from the mitochondrial matrix to the cytosol [48]. Consistent with this, our results show that MTFR2 binds to VDAC1 via the leucine 125 residue in the β8-strand to promote VDAC1 oligomerization, an effect abolished by L125A mutation or β8-strand truncation. While MTFR2 overexpression increases mitochondrial fragmentation, suppressing VDAC1 oligomerization via L125A mutation or inhibitor treatment reduces cytosolic mtDNA release and consequently attenuates M2 macrophage polarization and chemotaxis. Collectively, these results show that MTFR2 promotes pore formation that mediates mtDNA release into the cytosol by binding to the VDAC1 β8-strand via Leu125 and promoting VDAC1 oligomerization, thereby establishing a structural basis for metabolic regulation of macrophage function in the TME.

This study demonstrates for the first time that MTFR2 upregulates BCAT1 to reprogram BCAA metabolism. BCAT1 is a cytoplasm-localized BCAA metabolic enzyme whose transcription depends on nuclear transcription factor activation [49]. The mitochondrial localization of MTFR2 precludes direct binding to the BCAT1 promoter. Instead, MTFR2 may upregulate BCAT1 by inducing cytosolic mtDNA release. While cytosolic DNA can theoretically be recognized by multiple innate immune sensors, we found that TLR9 specifically mediated mtDNA recognition in this context, with minimal or negligible involvement of alternative sensors such as cGAS and NLRP3. As a specific PRR, TLR9 recognizes CpG-rich mtDNA [50] to activate the downstream NF-κB pathway, thereby enhancing BCAT1 transcription. These findings indicate MTFR2 initiates TLR9-NF-κB signaling via mitochondrial dysfunction to regulate BCAT1 transcription, consistent with its function as a mitochondrial fission regulator and revealing a potential mechanism for mitochondrial stress-mediated nuclear transcriptional activation.

BCAT1 catalyzes reversible conversion between BCAAs and BCKAs and mediates reversible transamination between BCAAs and α-ketoglutarate to generate glutamic acid [51, 52]. Within the TME, monocarboxylate transporter 1 (MCT1) mediates BCKA transport. Following uptake by TAMs, BCKAs are reconverted to BCAAs, reducing TAM phagocytic activity [16]. Depending on the metabolic context, BCKA family members exert heterogeneous effects on macrophage function. Macrophages can internalize KIC, KMV, and KIV secreted by tumor. KIC and KMV drive M2 macrophage polarization by enhancing the tricarboxylic acid cycle and polyamine metabolism, thereby impairing antitumor immunity [17, 18]. We observed that MTFR2 upregulates BCAT1 to promote KIC and KMV production, which activates the mTORC2-AKT signaling pathway in TAMs and drives M2 polarization. These findings support the role of BCKAs in TAM immunosuppression and reveal the MTFR2-BCAT1 axis as a targetable node to reverse LUAD-TAM crosstalk. Importantly, clinical analysis of LUAD specimens revealed that the positive correlation between MTFR2 and BCAT1 is preserved in patient tumors, underscoring its translational relevance and therapeutic potential for reversing immunosuppression in the TME.

As a key pathway for sensing amino acids, mTOR integrates metabolic signals to regulate cellular functions [53–55]. mTORC1 activation induces M1 marker genes (IL-1β, iNOS) by regulating HIF-1α and STAT1 [56, 57]. In contrast, mTORC2 phosphorylates AKT at Ser473 to activate downstream signals such as FOXO1 and GSK3β, promoting M2 marker expression (IL-10, Arg1) [58–60]. In this study, BCAT1 overexpression promotes BCKAs accumulation in the TME, with KIC and KMV driving M2 polarization via mTORC2-AKT signaling. Pharmacological blockade of AKT further confirmed the functional necessity of this pathway in mediating BCKA-induced macrophage polarization and pro-tumorigenic effects.

This study still has several limitations. First, although we validated the role of MTFR2 in malignant progression using three mouse models with different immune backgrounds, patient-derived xenograft models were not included. Additionally, the metabolic fates of KIC and KMV within macrophages require further investigation via isotope tracing and protein interactomics. The role of the abnormal BCAA mechanism in other immune cells, such as MDSCs and Tregs, remains unexplored. Impacts of BCAA deficiency on systemic metabolism and combination therapy strategies need more evaluation. Addressing these limitations in future studies will advance this mechanism toward clinical translation.

In summary, we identified a targetable bidirectional loop linking the MTFR2-VDAC1-BCAT1 axis in LUAD to the BCKAs-M2 axis in TAMs. Tumor-secreted KIC and KMV polarize macrophages to M2-TAMs, establishing continuously amplified positive feedback that promotes LUAD malignant proliferation and immune evasion. Based on this finding, targeting the MTFR2-BCAT1-TAMs axis has the potential to reduce M2-TAMs proportion and inhibit tumor growth.

## Materials and methods

### Cell line and reagents

The normal human lung epithelial cell BEAS-2B (RRID: CVCL_0168), human embryonic kidney cell HEK293T (RRID: CVCL_0063), mouse LUAD cell LLC (RRID: CVCL_4358), human LUAD cell lines A549 (RRID: CVCL_0023), H1299 (RRID: CVCL_0060), H1975 (RRID: CVCL_1511), H23 (RRID: CVCL_1547), PC9 (RRID: CVCL_B260) and human monocyte cell line THP-1 (RRID: CVCL_0006) were purchased from the National Collection of Authenticated Cell Cultures, and the cell lines were kindly confirmed to be contamination free. LUAD cell lines and THP-1 were cultured in Roswell Park Memorial Institute (RPMI)-1640 medium containing 10% fetal bovine serum (FBS) (Vazyme, F101) and 1% penicillin-streptomycin (NCM, C100C5) in an incubator at 37 °C with 5% CO_2_. BEAS-2B, HEK-293T, and LLC were cultured in Dulbecco’s Modified Eagle Medium (DMEM) medium supplemented with 10% FBS and 1% penicillin-streptomycin in an incubator at 37 °C with 5% CO_2_.

### Differentiation of macrophages

THP-1 were induced to M0 cells with 100 ng/mL phorbol-12-myristate-13-acetate (PMA) (MCE, HY-18739), and polarized to the M2 phenotype by 20 ng/mL interleukin-4 (Beyotime, P5129).

### Mice

Half female and half male mice (4–6 weeks old) weighing ≈18 g were obtained from GemPharmatech Co., Ltd. TheC57BL/6 mice, NCG mice, and BALB/c nude mice were maintained under specific pathogen-free conditions at the Laboratory Animal Center of Nanjing Medical University. All procedures were approved by the Experimental Animal Ethics Committee of Nanjing Medical University (No. IACUC-2410084).

### Clinical samples

Fresh clinical tissues were obtained from LUAD patients who underwent surgical resection at the First Affiliated Hospital of Nanjing Medical University. Following excision, a portion of the samples collected was used for organoids construction. The remaining samples were either preserved in 4% paraformaldehyde or kept at −80 °C for later research. The research protocol was reviewed and approved by the Institutional Review Board of Jiangsu Province Hospital (2023-SR-847). Informed consent was obtained from all patients. Patient information is provided in Supplementary Table 6.

### Tissue microarray (TMA) and Immunohistochemistry (IHC)

LUAD tissue microarray (TMA) (LUC1601 80A1) was obtained from Wuhan Bioqiandu Technology Co., LTD. A total of 80 pairs of lung adenocarcinoma (LUAD) tissues and adjacent normal tissues between January 2006 and December 2019 were collected to compose the TMA. All samples were pathologically confirmed, and clinical information (including gender, age, TNM stage, location, survival time, and status) was documented. The study was approved by the Institutional Ethics Committee (Approval No. XYLL-2021B001). Informed consent was obtained from all patients. Patient information is provided in Supplementary Table 7.

IHC staining was performed to detect MTFR2 and CD206 expression. Briefly, sections were dewaxed, rehydrated, and subjected to antigen retrieval. Primary antibodies against MTFR2 (Affinity, #DF14965), CD206 (abcam, #ab64693), and BCAT1 (Proteintech, 13640-1-AP) were incubated overnight at 4 °C, followed by HRP-conjugated secondary antibodies.

IHC analysis was performed using Aipathwell® software to calculate the histochemical score (H-score). The H-score is calculated as ∑(pi×i) = (percentage of weak intensity cells ×1) + (percentage of moderate intensity cells ×2) + (percentage of strong intensity cells ×3), where “i” represents the grading of positive area intensity: negative with no staining, scored as 0; weak positive with pale yellow staining, scored as 1; moderate positive with brownish-yellow staining, scored as 2; strong positive with brown staining, scored as 3. “pi” represents the percentage of positive area corresponding to each intensity grade. The H-score ranges from 0 to 300. Pearson correlation analysis was used to assess the relationship between MTFR2 and CD206 or BCAT1 expression.

### Public database analysis

RNA-seq data and the clinical information of 550 LUAD samples were downloaded from The Cancer Genome Atlas (https://portal.gdc.cancer.gov/), 14 samples were excluded due to incomplete clinical information or survival less than 30 days. In total, 536 samples, comprising 478 tumor samples and 58 healthy samples, were analyzed in the present study. Samples from two datasets, GSE31210 and GSE30219, were applied to validate the hub genes and risk score (https://www.ncbi.nlm.nih.gov/geo/). GSE31210 is annotated by GPL570 and includes 226 tumor samples and 20 healthy samples. GSE30219 is annotated by GPL570, comprising 293 tumor samples and 14 healthy samples; 4 samples were excluded due to incomplete clinical information or survival less than 30 days. Pearson correlation analysis was used to assess the relationship between MTFR2, CD206, BCAT1, and VDAC1 expression. Survival analysis was performed using Kaplan-Meier curves with log-rank tests.

### siRNAs/plasmids transfection and lentiviral infection

siRNA targeting specific genes were synthesized by Beijing Tsingke Biotechnology Co., Ltd. (see Supplementary Table 8 for sequences). MTFR2 and VDAC1 overexpression plasmids were constructed based on pLV3-CMV-MCS-3×FLAG-Puro, pCMV-MCS-3×Myc-Puro and pCMV-MCS-3×FLAG-Puro (Miaoling Biotechnology). Full-length (FL) VDAC1 and its truncation mutants were designed based on structural analysis. The truncation mutants included N-terminal truncation (N120, encoding amino acids 1–120), β8-strand-containing fragment (β8-SCF, encoding amino acids 100–220, covering β5–14 strands), and C-terminal truncation (C180, encoding amino acids 180–283). The VDAC1 Leu125Ala (L125A) point mutant was generated by site-directed mutagenesis, substituting leucine at position 125 with alanine. All VDAC1 constructs (FL, N120, β8-SCF, C180, L125A) were cloned into the 3×FLAG-tagged eukaryotic expression vectors (pLV3-CMV-MCS-3×FLAG-Puro or pCMV-MCS-3×FLAG-Puro). The MTFR2 gene was cloned into the pCMV-MCS-3×Myc-Puro vector. All constructs were verified by Sanger sequencing to ensure sequence accuracy. The shMTFR2 plasmids were generated using LV2-pGLV-u6-puro (Genepharma) (see Supplementary Table 8 for sequences). The siRNAs and plasmids were transfected into cells with Lipo8000™ Transfection Reagent (Beyotime, C0533).

Lentiviruses were produced by co-transfecting plasmids with packaging vectors (psPAX2 and pMD2.G) into HEK293T cells using Lipo8000™ Transfection Reagent. LUAD cells were infected with the viruses in the presence of 5 μg/mL polybrene for 48 h, followed by selection with 1–2 μg/mL of puromycin for 72 h. Overexpression or knockdown efficiency was verified by Western blot (Fig. S13).

### CCK-8

Cells were seeded in 96-well plates at 2000 cells per well. Then 10 µL CCK-8 reagent (Beyotime, C0037) was pipetted into each well containing 90 μL medium and incubated for 1–4 h in an incubator at 37 °C with 5% CO2 every 24 h for 4 days. Optical density (OD) values were measured at 450 nm by Multiskan FC microplate reader (Thermo Fisher, USA).

### Colony formation assay

Cells were seeded in 6-well plates at a density of 800 cells per well. After 1–2 weeks, cells were fixed with 4% paraformaldehyde and stained with 0.1% crystal violet for 10 min. Colonies were photographed by a digital camera and counted by ImageJ software.

### 3D tumor sphere formation assay

3D tumor spheres were constructed using U-bottom 96-well plates and 3D Culture Matrix (Beyotime, C0366S). The U-bottom 96-well plates were pre-coated with 40 μL 3D Culture Matrix per well and incubated at 37 °C for 10 min to form a thin coating layer. After aspirating the excess matrix solution, the culture plate is left to stand and dry in a laminar flow hood or biosafety cabinet for at least 10 min before cell seeding. LUAD cells were seeded at 2000 cells/well. Plates were centrifuged at 300 × *g* for 5 min to aggregate cells at the bottom center. After 7 days of culture, tumor spheres were observed and photographed under an inverted microscope (Olympus, Japan). The size and number of spheres (diameter > 50 μm) were analyzed using ImageJ software. Images of the Tumor Sphere were captured with an inverted microscope (Nikon TS2, Japan) every 24 h.

### 3D cell proliferation assay

Cell proliferation in 3D tumor spheres was evaluated using the CCK-8 (Beyotime, C0049S). 10 μL of CCK-8 solution was added to each well containing 3D spheres and incubated for 1–4 h in an incubator at 37 °C with 5% CO_2_ every 24 h for 4 days. OD values were measured at 450 nm by Multiskan FC microplate reader (Thermo Fisher, USA). The relative proliferation rate was calculated by normalizing the absorbance values of treated groups to the control group.

### Organoids culture

Rinse the tissue three times with 1 × D-PBS, which contains 2% antibiotics. Using surgical scissors, mince the tissue into little pieces measuring 1–3 mm^3^. Digest the tissue fragments with Gentle Cell Dissociation Reagent (Stemcell, #07174) at 37 °C for 30 min. 10% FBS to stop digestion, and filter utilizing a 70 μm cell strainer. Collect and centrifuge the filtered cells at 300 × *g* for 5 min at 4 °C. Aspirate the supernatant and resuspend the pellet in Matrigel® basement membrane matrix (Corning, 356234). The amount of Matrigel used depends on the size of the pellet. Approximately 4000 cells should be plated in 10 μL of Matrigel. Droplets of approximately 10 μL each are placed around the center of the well, and then place the culture plate into an incubator at 37 °C and 5% CO_2_ for 15–20 min to let the Matrigel solidify. Once the Matrigel droplets have solidified, add 100 μL of organoid complete medium (Mogengel bio, MA-0807T002SP) to each well.

### 3D cell and organoids viability (Live/Dead) staining

Live/dead staining was performed using the Calcein/PI/Hoechst Cell Viability Assay Kit (Beyotime Biotechnology, C1375S). 100 μL of staining solution (containing 1 μL 100X Calcein-AM and 100X M PI) was added to each well and incubated at 37 °C for 10 min in the dark. Stained spheres were visualized under a laser confocal microscopy (Leica, THUNDER DMi8). Live cells or organoids were stained green (Calcein-AM positive), and dead cells or organoids were stained red (PI positive). The live/dead ratio was analyzed using ImageJ software by quantifying the fluorescent area of each channel.

### Chemotaxis and co-culture assays

#### Chemotaxis assay

THP-1-derived macrophages (1 × 10⁵) were seeded in the upper chamber of Transwell inserts (LABSELECT, 14341, 8 μm pore size). Conditioned medium from LUAD cells under different treatment conditions was added to the lower chamber. After 24 h, migrated cells were fixed with 4% paraformaldehyde, and stained with 0.1% crystal violet for 10 min. Migrated cells were photographed by a digital camera and counted by ImageJ software.

#### Co-culture system

LUAD cell lines and THP-1-derived macrophages were co-cultured via a cell culture insert (LABSELECT, 14111, 0.4 μm pore size) to separate the upper and lower chambers. LUAD cells under different treatment conditions were seeded in the upper chamber, transferred to a 6-well plate that had previously been seeded with THP-1-derived macrophages. After 48 h of co-culture, macrophages were collected for flow cytometry or qPCR.

### Organoids co-culture system

LUAD cell lines and THP-1-derived macrophages were co-cultured via a cell culture insert (LABSELECT, 14511, 0.4 μm pore size) to separate the upper and lower chambers. LUAD cells under different treatment conditions were seeded in the upper chamber, transferred to a 6-well plate that had previously been seeded with THP-1-derived macrophages. After 48 h of co-culture, macrophages were collected for flow cytometry or qPCR.

### Cell-based conditional medium (CM)

For chemotaxis assay, LUAD-CM from LUAD cells, which were subjected to different treatment conditions, and fresh medium (1:1) were placed in the lower chambers of the Transwell system. For the functional study of LUAD cells, CM generated from the co-culture system (upper: LUAD cells under different treatment conditions; lower: THP-1-derived macrophages) were added into the medium of LUAD cells.

### RNA extraction and quantitative real-time PCR (qRT-PCR)

Total RNA was extracted from treated cells using an RNA extraction kit (Beyotime, China), followed by reverse transcription to complementary DNA (cDNA) with HiScript III RT SuperMix for qPCR (Vazyme, China) with 1 μg of total RNA. Amplification of synthesized cDNA was performed using HiScript III RT SuperMix for qPCR (Vazyme, China). ACTB was used as an internal control to normalize gene expression. The relative RNA levels of the indicated genes were calculated using the 2^−ΔΔCT^ method. Each experiment was performed in triplicate. The primer sequences are listed in Supplementary Table 9.

### Western blot and co-immunoprecipitation (Co-IP)

#### Western blot

Cells were lysed in Cell lysis buffer for Western and IP (Beyotime, P0013J) containing protease and phosphatase inhibitors. Protein concentration was quantified using BCA Protein Assay Kit (Beyotime, P0012). Equal amounts of protein were separated by SDS-PAGE, transferred to PVDF membranes, and probed with primary antibodies against MTFR2 (Proteintech, 26569-1-AP), BCAT1((Proteintech, 13640-1-AP), COXIV (Proteintech, 11242-1-AP), DNM1L (Proteintech, 12957-1-AP), VDAC1 (Proteintech, 66345-1-Ig), P65 (Proteintech, 10745-1-AP), P-P65 (Ser536, Beyotime, AF5881), AKT (Bioss, bsm-63324R), P-AKT(Ser473) (Litho, LTO6000P), LAMINB1(Selleck, F0523), TUBULIN (Proteintech, 66031-1-Ig), GAPDH (Servicebio, GB11002-100). HRP-conjugated secondary antibodies were used, and bands were visualized with ECL reagents (GLPBIO). The antibody information is listed in Supplementary Table 10. The original bright-field images of Western blot are provided in Fig. S14.

#### Co-IP

Cells were lysed in Cell lysis buffer for Western and IP (Beyotime, P0013J) with inhibitors. Lysates were incubated with anti-MTFR2 antibody (Proteintech, 26569-1-AP), VDAC1 (Proteintech, 66345-1-Ig) or IgG control overnight at 4 °C, followed by Protein A/G agarose (Santa, SC-2003) for 2 h. Immunoprecipitated proteins were analyzed by Western blot.

### Cellular fractionation

#### Cytoplasmic/nuclear Isolation

Cytoplasmic and nuclear fractions were isolated using the Nuclear and Cytoplasmic Protein Extraction Kit (Beyotime, P0027). Add 200 µL cytoplasmic isolation buffer A to 2 × 10^6^ cells, vortex at maximum speed for 5 s, then incubate on ice for 10 min. Add 10 µL cytoplasmic isolation buffer B, vortex at maximum speed for 5 seconds, then incubate on ice for 1 min, and centrifuge at 16,000 × *g* for 5 min at 4 °C to collect the supernatant as cytoplasmic Protein. Add 50 μL nuclear extraction buffer to the pellet, vortex at maximum speed for 30 s. Then incubate on ice, and vortex at maximum speed for 30 s every 2 min, totaling 30 min. Centrifuge at 16,000 × *g* at 4 °C for 10 min. The supernatant is the extracted nuclear protein.

#### Mitochondria/cytoplasm Isolation

Mitochondria and cytoplasm were separated using the Cell Mitochondria Isolation Kit (Beyotime, C3601). Add 500 µL isolation buffer to 1 × 10^7^ cells, incubate on ice for 10 min, then transfer the cell suspension to a glass homogenizer and homogenize approximately 15 times. Centrifuge cell homogenate at 1000 × *g*, 4 °C for 10 min, then transfer supernatant to a new tube, centrifuge at 3500 × *g*, 4 °C for 10 min. The pellet is high-purity mitochondria. Centrifuge the supernatant at 12,000 × *g*, 4 °C for 10 min. The supernatant is mitochondrial-depleted cytoplasmic proteins.

Purity of fractions was verified by Western blot using specific markers: COXIV (mitochondria), TUBULIN (cytoplasm), and LAMINB1 (nucleus).

### Proteinase K protection assay

high-purity mitochondria were extracted by Cell Mitochondria Isolation Kit from A549 and H1299 cells. Incubate mitochondria with 0, 0.5, 1, 2, 5, 10, and 20 μg/ml protease K (Beyotime, ST533) respectively for 20 minutes for digestion. Then analyze the localization of MTFR2 by Western blot. The outer mitochondrial membrane (OMM) proteins VDAC1 and DNM1L, and the inner mitochondrial membrane (IMM) protein COXIV serve as markers for OMM and IMM, respectively.

### Cytosolic mtDNA isolation and quantification

High-purity cytoplasm fractions without Mitochondria were extracted by Cell Mitochondria Isolation Kit from A549 and H1299 cells. Extract DNA components from cytoplasm or Whole cell lysate using the TIANamp Genomic DNA Kit (TIANGEN, YDP304-03). Then, RT-qPCR assays were used to detect mtDNA levels. Amplify mitochondrial DNA (mtDNA) using ND1 and D-LOOP primers, and amplify nuclear DNA (nDNA) using TERT and 18 s primers for qPCR analysis. Use TERT and 18 s as internal reference. The primer sequences are listed in Supplementary Table 9.

### DNase I treatment

To digest the cytosolic mtDNA, PULSin™ protein delivery reagent (Polyplus Transfection, 501-01) was used to deliver DNase I according the manufacturer’s instructions. LUAD cells cells were washed three times using serum-free RPMI-1640 and transfected with DNase I/PULSin mixture containing 3 μg DNase I for 4 h at 37 °C. Then, removing the mixture and the cells were cultured with complete medium for another 24 h for further studies.

### VDAC1 cross-linking assay

The VDAC1 cross-linking assay was performed as previously reported with modifications [44, 61]. Briefly, cells were harvested and washed twice with cold PBS (pH 8.0), followed by centrifuging at 300 × *g* for 5 min at 4 °C. The collected pellets were resuspended in 200 μL of 0.5 mM Ethylene glycol-bis (EGS, aladdin, E294679) solution diluted with cold PBS (pH 8.0), and incubated for 30 min at 30 °C. To quench the reaction, 2.5 μL of 1 M Tris-HCL (pH 7.5) was added, and the reaction mixture was cultured at 30 °C for 15 min. Finally, the treated cells were lysed in lysis buffer and the protein concentration was quantified using BCA Kit. 50 μg of the protein samples were subjected to 4–12% gradient SDS-page and immunoblotting using an anti-VDAC1 antibody.

### Flow cytometry

#### Phenotypic analysis of THP-1-induced macrophages

Cells were resuspended in staining buffer (Proteintech, PF00018) and adjusted to a density of 1 × 10^7^ cells/mL. Aliquots of 100 µL cell suspension were transferred to round-bottom polypropylene tubes. For cell surface marker staining, anti-CD68-594 (abclonal, A24176) and anti-CD86-APC (abclonal, A26126) were added, followed by incubation in the dark at room temperature for 30 min. Samples were then resuspended by flow cytometry fix buffer (Proteintech, PF00016) and permeabilized by flow cytometry perm buffer (Proteintech, PF00017). Intracellular staining was performed with anti-CD206-FITC (Elabscience, E-AB-F1161C) in the dark at room temperature for 30 min, followed by three washes with PBS. Fluorescence signals were detected using a Beckman flow cytometer and analyzed with FlowJo software.

#### Analysis of the tumor immune microenvironment

Tumor tissues of equal weight from different groups were harvested, immersed in buffer, and minced into small pieces. For tissue dissociation, 100 µg/mL DNase I (Biosharp, BS137) and 1 mg/mL collagenase IV (Biosharp, BS165) were added to the tumor homogenate, which was then incubated at 37 °C for 1 h. The digestion was terminated by adding PBS containing 10% FBS. The tumor cell suspension was filtered through a 70 µm cell strainer, centrifuged, and resuspended in pre-cooled staining buffer (BD, USA) to a density of 1 × 10^7^ cells/mL. A 100 µL aliquot of the cell suspension was transferred to round-bottom polypropylene tubes. After adding 1 µL of anti-mouse CD16/CD32 antibody for 10 min on ice, then stained with anti-F4/80-488 (abclonal, A27479), anti-CD206-PE (eBioscience, 12-2061-82), and anti-CD86-APC (abclonal, A27138) in the dark for 30 min at room temperature, followed by three PBS washes. 7AAD staining was subsequently performed. Fluorescence was detected using a Beckman flow cytometer and analyzed with FlowJo software.

### Mitotracker, TMRM, ROS, and Hoechst staining

Cells were stained with Mitotracker (invitrogen, M22426), Tetramethylrhodamine (TMRM) (invitrogen, I34361), ROS (Beyotime, S0033S) and Hoechst 33342 (Yeasen, 40731ES10) yielding a final concentration of 200 nM for 30 min, 100 nM for 30 min, 10 µM for 20 min, 0.5 µg/mL for 10 min respectively. The cells were then collected and washed with PBS three times. Fluorescence in live cells was captured by multimodal super-resolution live cell microscope (Nanoinsights, Multi-SIM) or confocal microscopy (Leica, STELLARIS 5), and detected using a flow cytometer (Beckman, Cytoflex S).

The average branch length, average area, and aspect ratio of mitochondria in cells were quantified using the Mitochondria Analyze plugin of ImageJ software.

### mtDNA localization

Cells were sequentially stained with PicoGreen (Yeasen, 12641ES, 3 µL/mL, 1 h), MitoTracker (Invitrogen, M22426, 200 nM, 30 min), and Hoechst 33342 (Yeasen, 40731ES10, 0.5 µg/mL, 10 min) in living cells. After washing with PBS, cells were fixed with 4% paraformaldehyde for 15 min, permeabilized, and immunostained with anti-TFAM antibody (Abclonal, A23467, 1:100) to specifically label mtDNA. Images were acquired using a multimodal super-resolution microscope (Nanoinsights, Multi-SIM). Cytosolic dsDNA was analyzed using ImageJ with the Coloc 2 plugin. Pearson correlation coefficients between TFAM and PicoGreen signals were calculated in cytoplasmic regions outside MitoTracker-positive mitochondrial contours (excluding Hoechst-positive nuclei) to confirm mtDNA identity.

### Immunofluorescence

Cells were fixed with 4% paraformaldehyde, permeabilized with 0.1% Triton X-100, and blocked with 5% goat serum. Primary antibodies against MTFR2 (Proteintech, 26569-1-AP), VDAC1 (Proteintech, 66345-1-Ig), TFAM (Abclonal, A23467), P65 (Proteintech, 10745-1-AP), TLR9 (CST, #13674), cGAS (Proteintech, 26416-1-AP), NLRP3 (Proteintech, 30109-1-AP), and anti-DNA (PROGEN, 61014_1) were incubated overnight at 4 °C, followed by Alexa Fluor-conjugated secondary antibodies (Beyotime). Images were captured by confocal microscopy (Leica, STELLARIS 5). Nuclear DNA was stained with DAPI (Beyotime, C1005) in blue.

### Proximity ligation assay (PLA)

Cells were fixed with formaldehyde and then permeabilized using ice-cold 90% methanol. Sample preparation was performed using the Duolink® PLA (Merck, DUO92008) strictly following the manufacturer’s protocol. Primary antibodies against MTFR2 (Proteintech, 26569-1-AP) and VDAC1 (Proteintech, 66345-1-Ig), Flag (Elabscience, E-AB-48025), and Myc (CST, 2278) were used, with the amplification reaction time setting at 150 min.

### Chromatin immunoprecipitation (ChIP)-qPCR

Chromatin immunoprecipitation was performed using the ChIP Assay Kit (Beyotime, P2080S): Cells were cross-linked with 1% formaldehyde for 10 min at room temperature and quenched with glycine; cells were lysed, and chromatin was sheared into 200–500 bp fragments by sonication; Lysates were immunoprecipitated with anti-P65 antibody (Proteintech, 10745-1-AP) or IgG control. Precipitated DNA was purified and analyzed by agarose gel electrophoresis or quantitative PCR with specific primers. The primer sequences are listed in Supplementary Table 9.

### Mass spectrometry analysis

Co-IP proteins were visualized by Coomassie Blue Staining Kit (Beyotime, P0017A). The protein bands of interest were excised from the gel and subjected to in-gel trypsin digestion. Resulting peptides were extracted and analyzed by nanoLC-MS/MS (Sangon Biotech, Shanghai, China) using an Orbitrap Fusion mass spectrometer. The shotgun strategy was employed for proteomics studying. Protein identification was performed using Mascot search engine and the SwissProt database. The mass spectrometry proteomics data have been deposited to the ProteomeXchange Consortium (http://proteomecentral.proteomexchange.org) via the iProX partner repository [45, 62] with the dataset identifier PXD067030.

### Metabolomics analysis

Collect the culture supernatants of A549-Vector and A549-MTFR2 cells, and centrifuge at 2000 × *g*, 4 °C for 15 min to remove cells. Centrifuge the separated supernatant at 12,000 × *g*, 4 °C for 10 min to further remove impurities such as cell debris. Then take the supernatant and further concentrate it to 500 μl. To extract metabolites from samples, 400 μL of cold methanol/acetonitrile (1:1, v/v) extraction solvent was added to remove the protein and extract the metabolites, then adequately vortexed. For absolute quantification of the metabolites, stock solutions of stable-isotope internal standards were added to the extraction solvent simultaneously. The mixture was collected into a new centrifuge tube, and centrifuged at 14,000 × *g* for 20 min at 4 °C to collect the supernatant. The supernatant was dried in a vacuum centrifuge. For LC-MS analysis, the samples were re-dissolved in 100 μL acetonitrile/water (1:1, v/v) solvent and centrifuged at 14,000 × *g* at 4 °C for 15 min, then the supernatant was injected. Prepared samples were analyzed by LC-MS. Peak extraction was performed on MRM raw data using MultiQuant or Analyst software to obtain the ratio of the peak area of each substance to the peak area of the internal standard, and the content was calculated according to the standard curve (Supplementary Table 11). Secondary mass spectrometry (MS/MS) spectra of major metabolites were showed in Fig. S9.

### RNA-sequencing (RNA-seq)

RNA-seq was performed using the Illumina PE150 instrument by KAITAI-BIO (China, Hangzhou). Alignment and transcript splicing analyses were conducted using Hisat2 (http://ccb.jhu.edu/software/hisat2) and Stringtie (https://ccb.jhu.edu/software/stringtie/), respectively. The fragments per kilobase million values were used for quantitative analysis of all genes, and differentially expressed genes were identified with adjusted *p* value < 0.05 and |log2(FoldChange)| > 1. The differentially expressed genes were subjected to Gene Set Enrichment Analysis (GSEA) and Kyoto Encyclopedia of Genes and Genomes pathway (KEGG) analysis. Sufficient replicates were conducted to ensure statistical significance of the analysis. The full RNA-seq dataset was uploaded to the NCBI Sequence Read Archive (SRA) database (accession code: PRJNA1302298).

### Animal experiments

#### Xenograft experiments

Cancer cells(2 × 10⁶) under different treatments were resuspended in PBS and injected into nude mice subcutaneously. Tumor volumes and nude mice weight were measured every 2 days. Once tumors reached a diameter of ≈3 mm, mice were treated with vehicle (diluted solution: 5% DMSO + 40% PEG300 + 5% Tween80 + 50% ddH2O or ddH2O), or BCATc Inh2 (30 mg/kg) (MCE, HY-116044) by intraperitoneal injection every two days for 14 days. The tumor volume was quantified using the formula of length times the square of width times 0.5. The length was also considered as the diameter here. The maximal tumor burden permitted by the ethics committee is a length of 1.5 cm, and the maximal tumor burden did not exceed the limit.

#### Macrophage depletion

Mice were injected with clodronate liposomes (1 mg/200 μL) (Yeasen, 40337ES08) via the tail vein to deplete macrophages.

#### Immunohistochemistry (IHC) and multiplex immunofluorescence

Animal tumor tissues were fixed with 4% paraformaldehyde, then paraffin-embedded and sectioned. The slides were deparaffinization, rehydration, and antigen retrieval before being blocked with 5% BSA (Sigma-Aldrich, USA) for 1 h. The primary antibodies were then incubated with the slides overnight at 4 °C. The slides were treated with 3,3′- Diaminobenzidine (Sigma-Aldrich, USA) and hematoxylin (Sigma-Aldrich, USA) after the secondary antibodies were incubated for 30 min at room temperature. Images of immunohistochemistry were obtained at random under a microscope, and the outcomes were examined using ImageJ software.

For multiplex immunofluorescence, the slides were deparaffinization, rehydration, and antigen retrieval before being blocked with 10% donkey serum for 30 min. The primary antibodies were then incubated with the slides overnight at 4 °C. The slides were treated with Tyramine salt-CY3/CY5/488/594/450 sequentially after the secondary antibodies (HRP enzyme labeled) were incubated for 50 min at room temperature. Images of mIHC were obtained at random under a fluorescence microscope. Nuclei stained with DAPI were blue under UV excitation and positive signals were labeled with the corresponding fluorescein. Positive signal intensity in the tumor tissue area was quantified separately for each section using ImageJ software.

### Statistical analysis

All statistical analyses were performed utilizing R statistical software (version 4.3.3; R Foundation for Statistical Computing) and GraphPad Prism 8 (GraphPad Software Inc.). Data are presented as mean ± SEM. Continuous variables with normal distribution were compared between groups using Student’s independent *t*-test, while categorical variables were analyzed through Pearson’s chi-square test. Nonparametric Spearman’s rank correlation coefficient was employed to assess bivariate associations between continuous variables. Survival outcomes were evaluated through Kaplan–Meier methodology with log-rank testing for univariate analysis, followed by multivariable Cox proportional hazards regression modeling to adjust for potential confounding factors. All statistical tests were conducted using two-tailed comparisons, with a probability threshold of *P* < 0.05 defining statistical significance. All experiments were repeated at least three times.

## Supplementary information

Supplementary Figures and legends

Supplementary Fig. S14-Original bright-field images of Western Blot

Supplementary Table 1

Supplementary Table 2

Supplementary Table 3

Supplementary Table 4

Supplementary Table 5

Supplementary Table 6

Supplementary Table 7

Supplementary Table 8

Supplementary Table 9

Supplementary Table 10

Supplementary Table 11

## Data Availability

The data that support the findings of this study are available in the supplementary material of this article.

## Code availability

The R code used to analyze the data in this paper is available upon reasonable request. Interested investigators may contact the corresponding author for access to the code.
