# Supplementary material for: MTFR2 promotes VDAC1 oligomerization to reprogram BCAA metabolism in tumor cells to polarize TAMs in LUAD
Source: Cell Death Dis. 2026 May 20;17(1):710. doi: 10.1038/s41419-026-08871-2 (PMC13462101; doi:10.1038/s41419-026-08871-2)

**Fig. S1D**

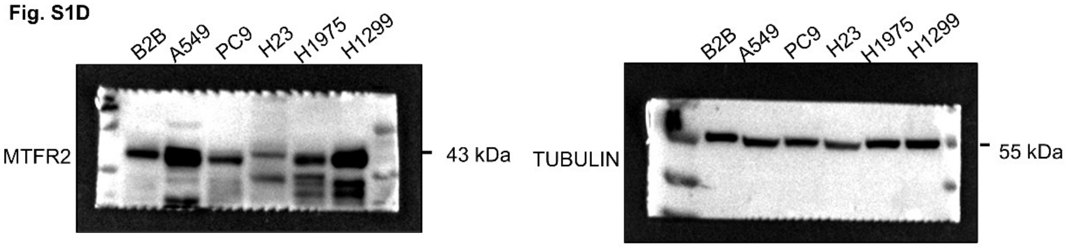


**Fig. S5B**

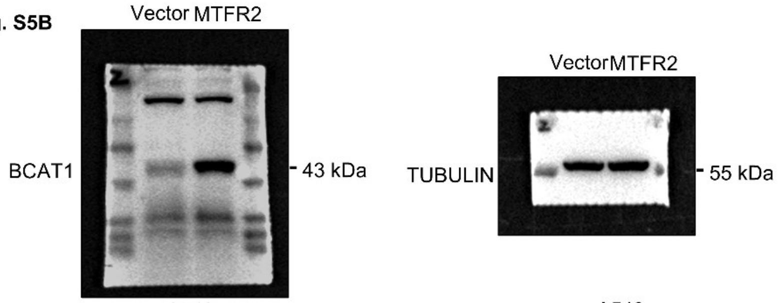


**Fig. S5B**

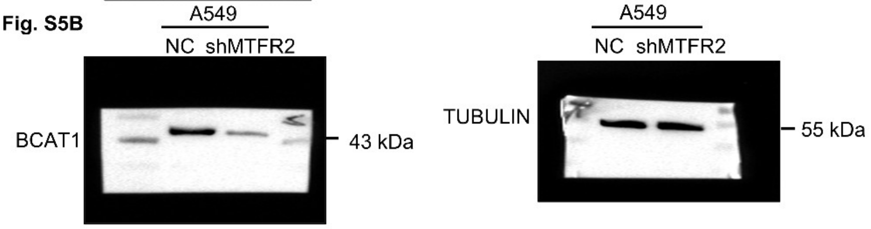


**Fig. 3A**

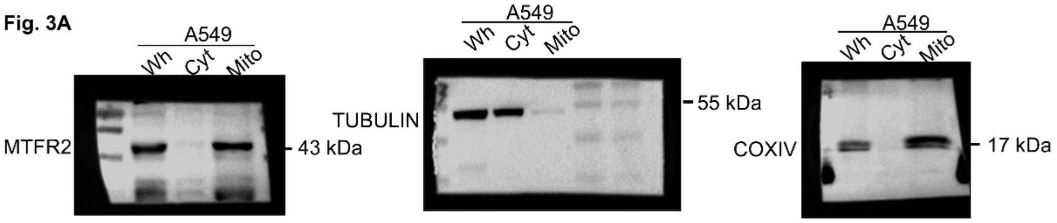


**Fig. 3B**

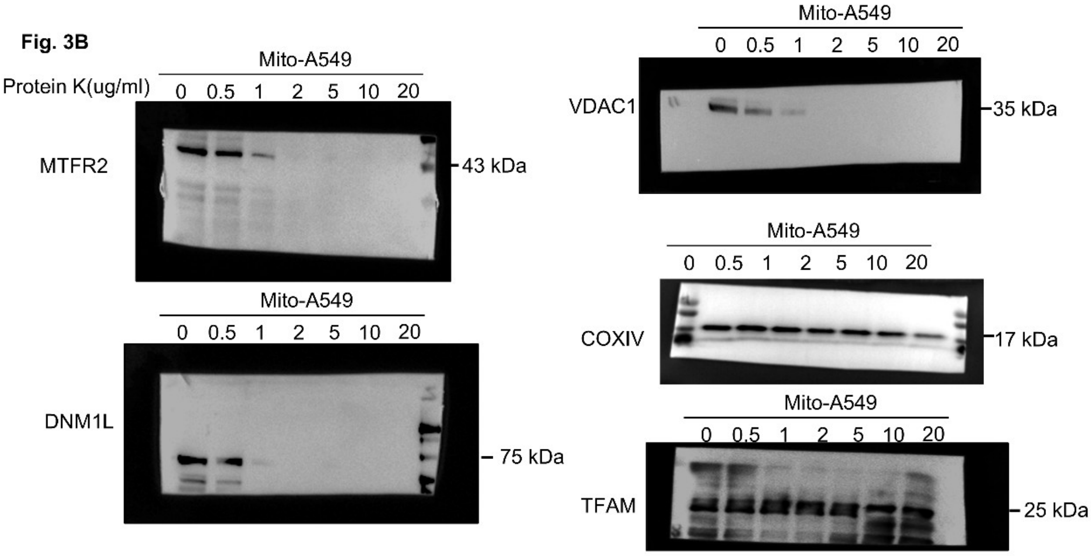

Fig. S6A

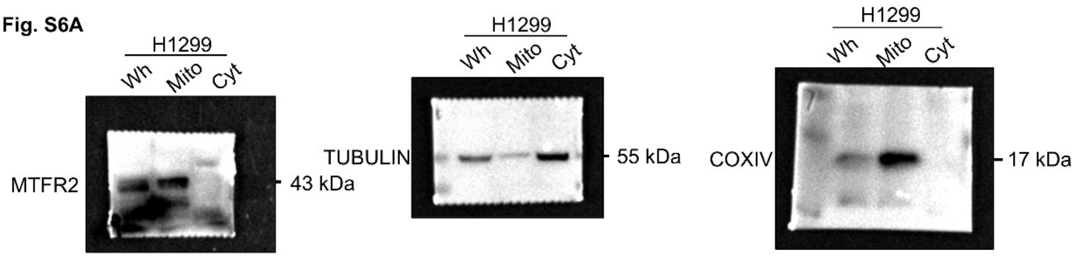


Fig. S6B

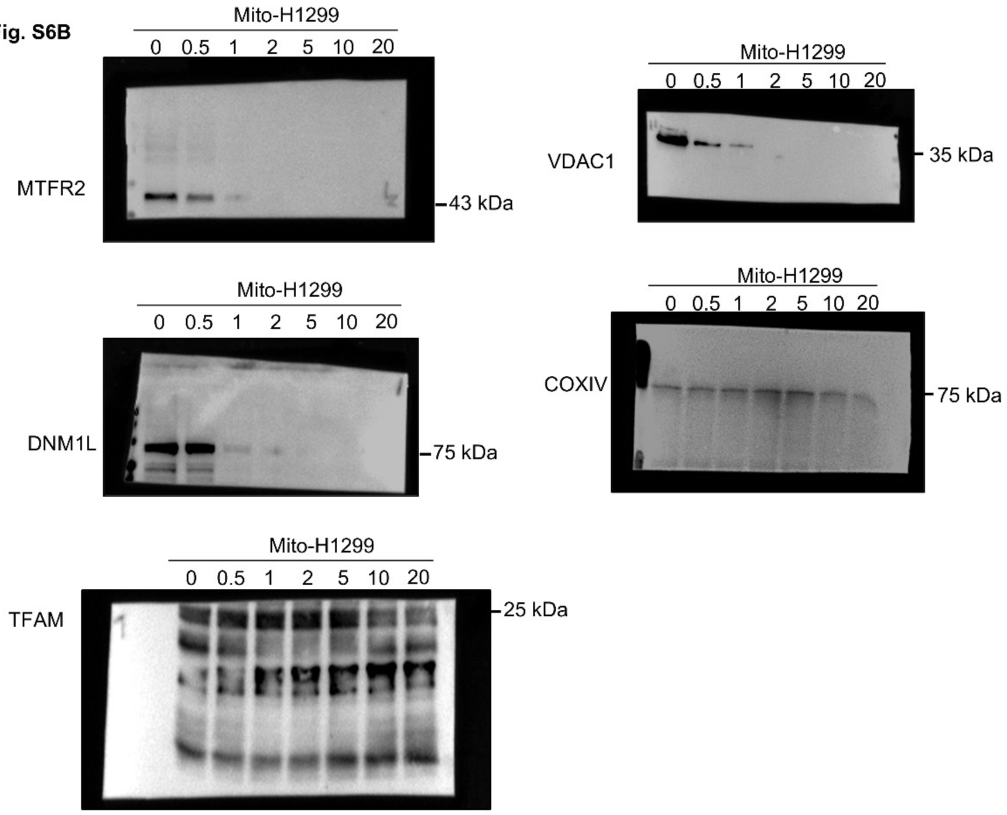

**Fig. 4B**

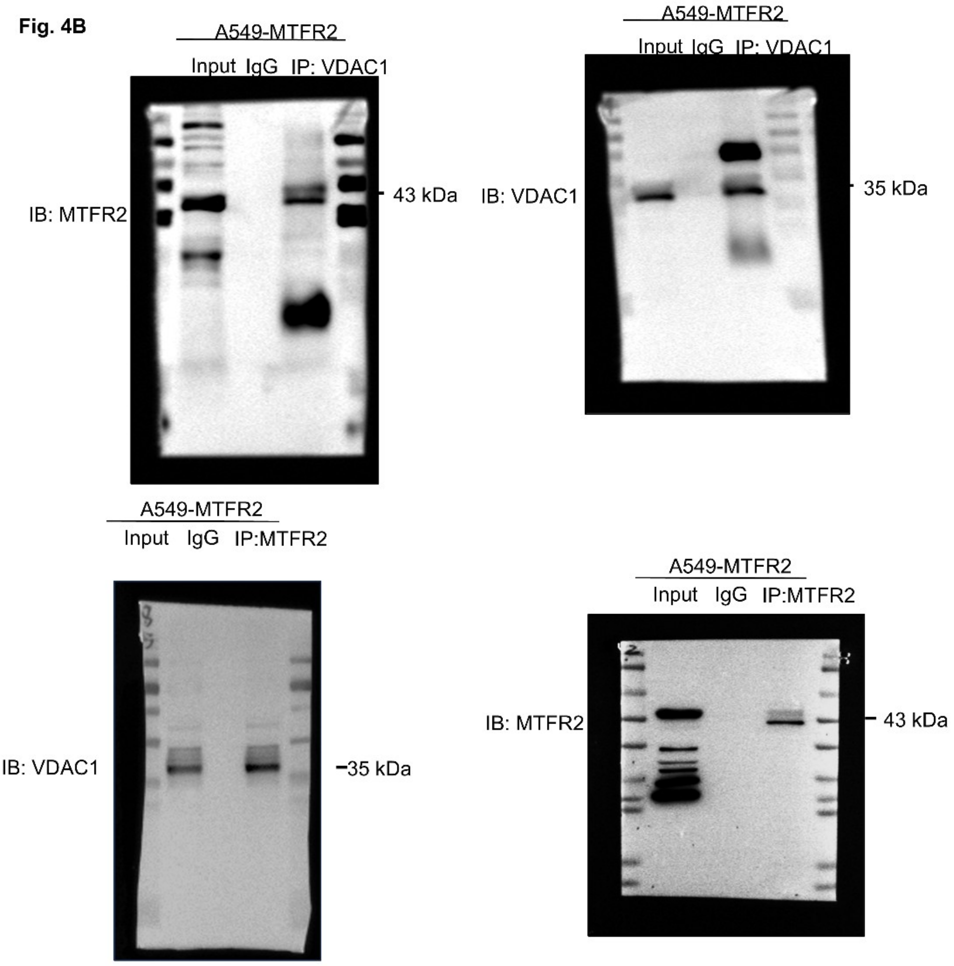
A549-MTFR2
Input IgG IP: VDAC1
IB: MTFR2
43 kDa
A549-MTFR2
Input IgG IP: VDAC1
IB: VDAC1
35 kDa
A549-MTFR2
Input IgG IP:MTFR2
IB: VDAC1
35 kDa
A549-MTFR2
Input IgG IP:MTFR2
IB: MTFR2
43 kDa

Fig. 4G

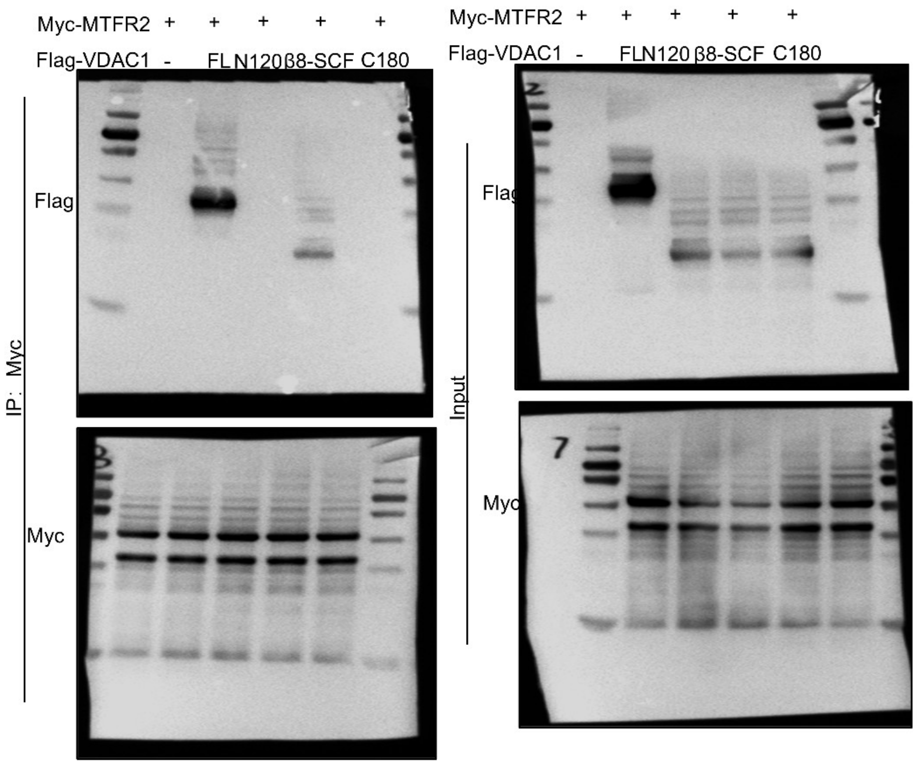

Myc-MTFR2 + + + + +
Flag-VDAC1 - FL N120 β8-SCF C180
Flag
Myc
IP: Myc
Myc-MTFR2 + + + + +
Flag-VDAC1 - FL N120 β8-SCF C180
Flag
Myc
Input


Fig. 4H

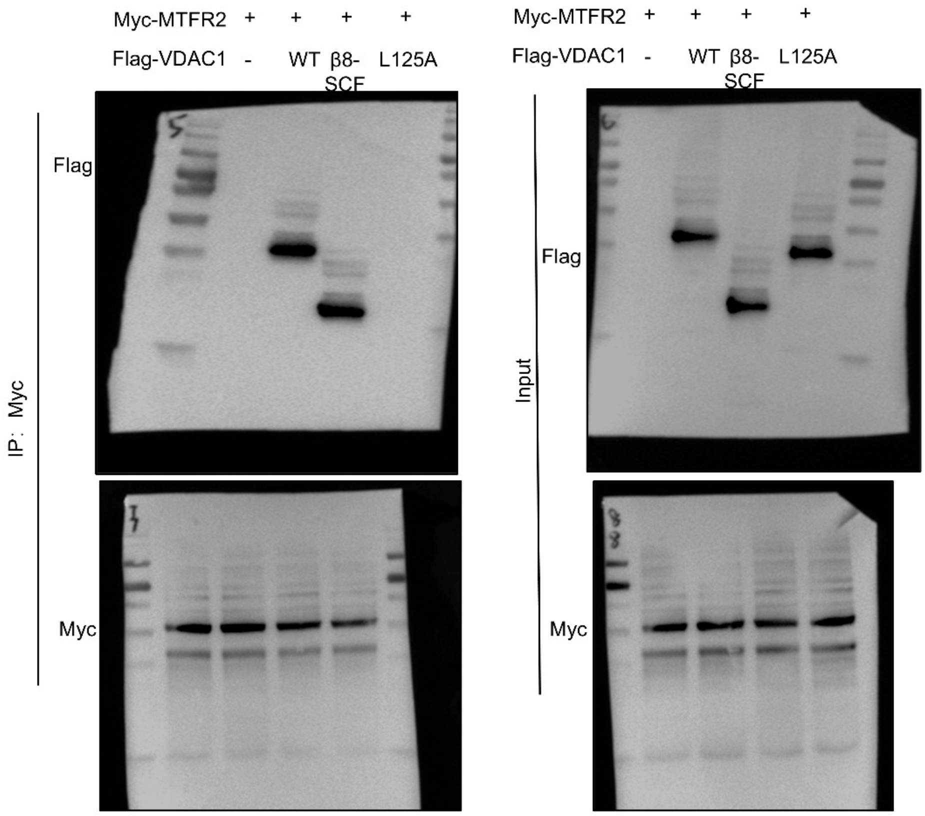

Myc-MTFR2 + + + +
Flag-VDAC1 - WT β8-SCF L125A
Flag
Myc
IP: Myc
Myc-MTFR2 + + + +
Flag-VDAC1 - WT β8-SCF L125A
Flag
Myc
Input

**Fig. 5A**

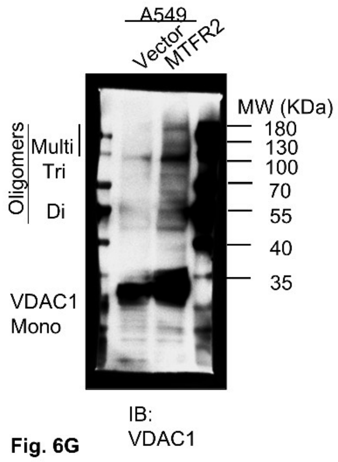


**Fig. 5B**

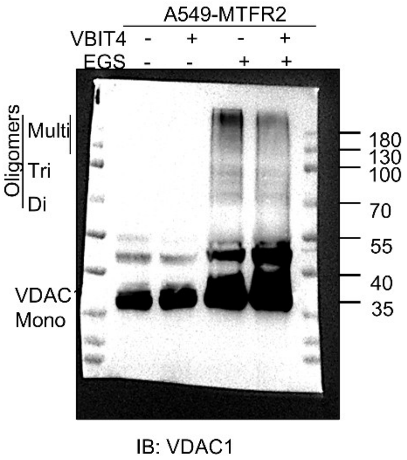


**Fig. 5C**

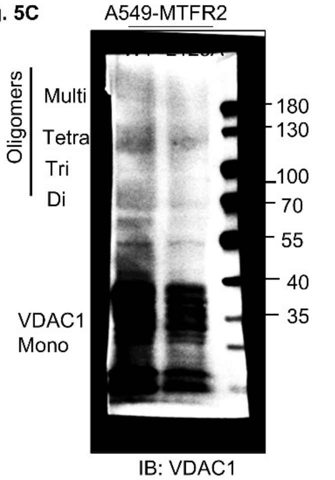


**Fig. 6G**

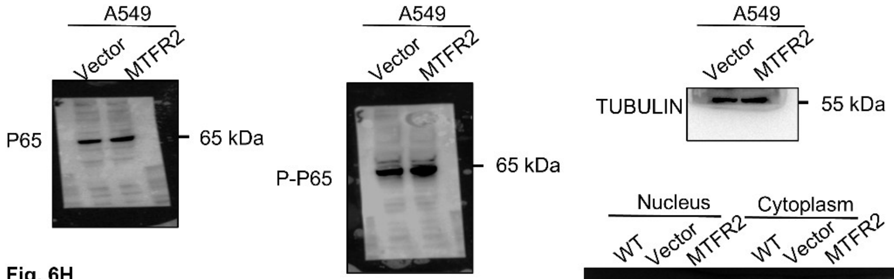


**Fig. 6H**

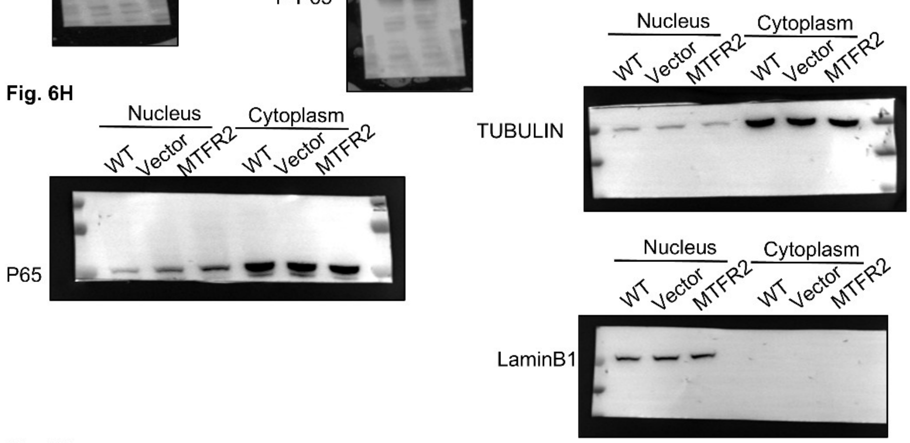


**Fig. 7K**

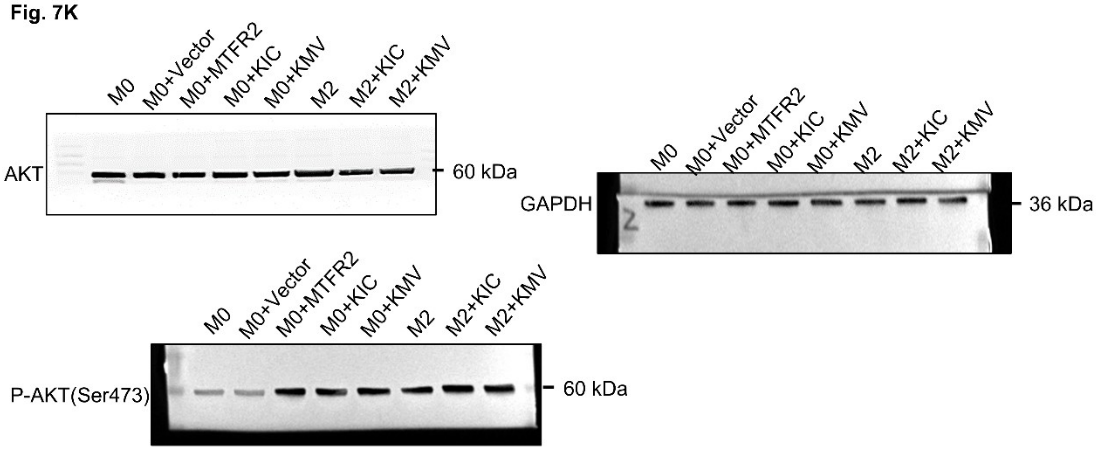

**Fig. S13A**

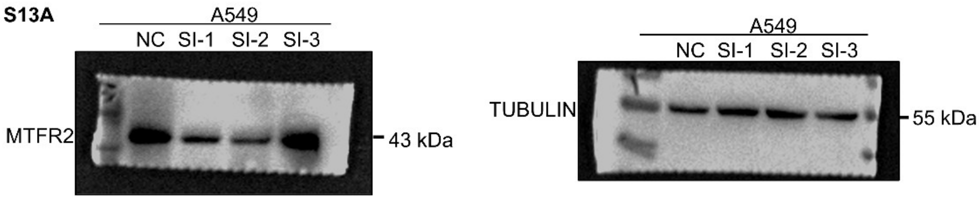


**Fig. S13B**

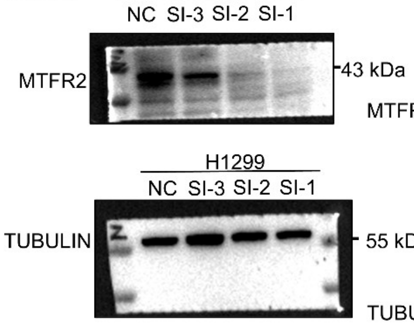


**Fig. S13C**

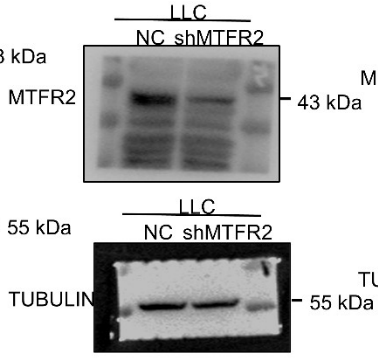


**Fig. S13D**

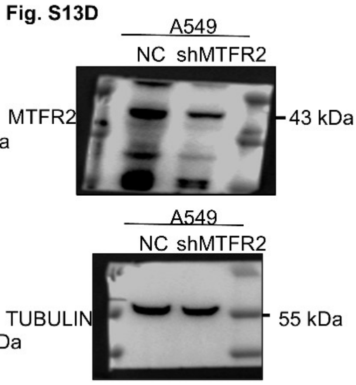


**Fig. S13E**

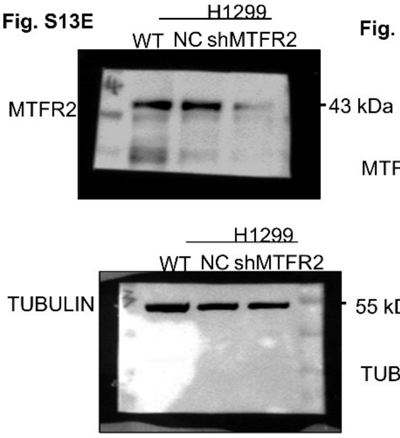


**Fig. S13F**

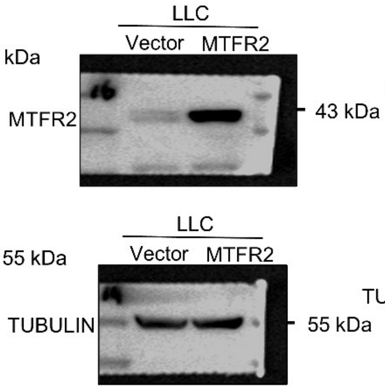


**Fig. S13G**

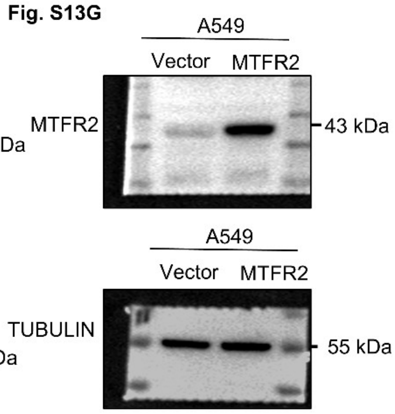


**Fig. S13H**

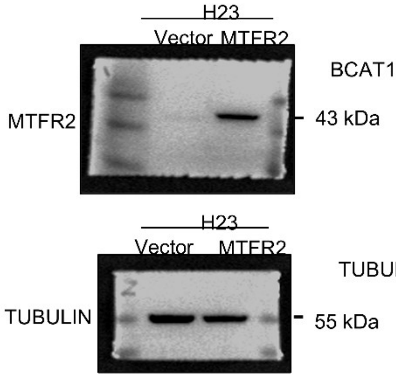


**Fig. S13J**

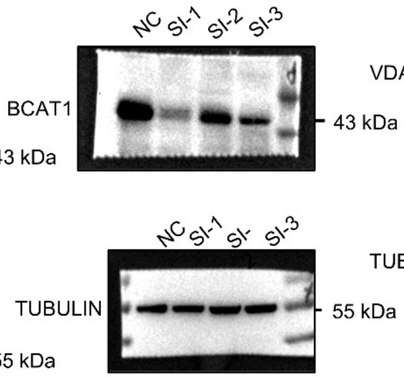


**Fig. S13K**

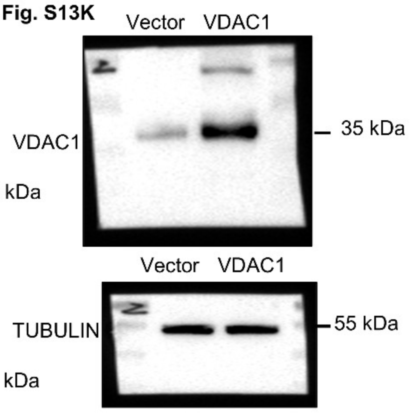

Supplement: Supplementary file 1 [file 41419_2026_8871_MOESM2_ESM.docx]
