# Supplementary material for: MTFR2 promotes VDAC1 oligomerization to reprogram BCAA metabolism in tumor cells to polarize TAMs in LUAD
Source: Cell Death Dis. 2026 May 20;17(1):710. doi: 10.1038/s41419-026-08871-2 (PMC13462101; doi:10.1038/s41419-026-08871-2)
Supplement: Supplementary file 1 [file 41419_2026_8871_MOESM10_ESM.docx]

**Supplementary Table 8. List of siRNA and shRNA sequences.**

| **siRNAs** | **sense（5′-3′）** |
| --- | --- |
| SiMTFR2#1 | GCGGUAGACCCAUUCAUAA |
| SiMTFR2#2 | GUACAACCAGGAUCUAAUA |
| SiMTFR2#3 | GUGGAUCUAUGGUUCCAUCUU |
| SiBCAT1#1 | GCGAGAGACACAUCACCAU |
| SiBCAT1#2 | GAGCCCAGUGGGACCUUAUUU |
| SiBCAT1#3 | GGCUGCAACUAUGGAUCUU |
| **shRNA** | **sense（5′-3′）** |
| ShMTFR2#2 | GTACAACCAGGATCTAATA |
