# Supplementary material for: MTFR2 promotes VDAC1 oligomerization to reprogram BCAA metabolism in tumor cells to polarize TAMs in LUAD
Source: Cell Death Dis. 2026 May 20;17(1):710. doi: 10.1038/s41419-026-08871-2 (PMC13462101; doi:10.1038/s41419-026-08871-2)
Supplement: Supplementary file 2 [file 41419_2026_8871_MOESM11_ESM.docx]

**Supplementary Table 9. List of primer sequences for qPCR.**

| **Oligo Name** | **Sequence（5′-3′）** |
| --- | --- |
| ACTB qF | CACCATTGGCAATGAGCGGTTC |
| ACTB qR | AGGTCTTTGCGGATGTCCACGT |
| MTFR2 qF | TGCTTCCACTGGAACCTTGTCG |
| MTFR2 qR | CGAAATCTGAGATAACTGGCTTCT |
| CD68 qF | CGAGCATCATTCTTTCACCAGCT |
| CD68 qR | ATGAGAGGCAGCAAGATGGACC |
| CD86 qF | CCATCAGCTTGTCTGTTTCATTCC |
| CD86 qR | GCTGTAATCCAAGGAATGTGGTC |
| iNOS qF | GCTCTACACCTCCAATGTGACC |
| iNOS qR | CTGCCGAGATTTGAGCCTCATG |
| CD163 qF | CCAGAAGGAACTTGTAGCCACAG |
| CD163 qR | CAGGCACCAAGCGTTTTGAGCT |
| CD206 qF | AGCCAACACCAGCTCCTCAAGA |
| CD206 qR | CAAAACGCTCGCGCATTGTCCA |
| Arg1 qF | TCATCTGGGTGGATGCTCACAC |
| Arg1 qR | GAGAATCCTGGCACATCGGGAA |
| ND1 qF | CACCCAAGAACAGGGTTTGT |
| ND1 qR | TGGCCATGGGTATGTTGTTAA |
| D-LOOP qF | CTATCACCCTATTAACCACTCA |
| D-LOOP qR | TTCGCCTGTAATATTGAACGTA |
| TERT qF | TCACGGAGACCACGTTTCAAA |
| TERT qR | TTCAAGTGCTGTCTGATTCCAAT |
| 18s qF | TAGAGGGACAAGTGGCGTTC |
| 18s qR | CGCTGAGCCAGTCAGTGT |
| BCAT1 qF | TGAGGCTTGGCTTTTGTGAA |
| BCAT1 qR | GGCTCTGGTGTAACAAAGCC |

**Primers for ChIP-qPCR**

| **Oligo Name** | **Sequence（5′-3′）** |
| --- | --- |
| BCAT1 Site1 qF | AGGGAACTTACAGGGGACCA |
| BCAT1 Site1 qR | GAACATGTGCCTGGTTTGCC |
| BCAT1 Site2 qF | CCCAAAGTGCTGAGATTA |
| BCAT1 Site2 qR | AACTGAGGAATGGCTAAA |
