# Supplementary material for: MTFR2 promotes VDAC1 oligomerization to reprogram BCAA metabolism in tumor cells to polarize TAMs in LUAD
Source: Cell Death Dis. 2026 May 20;17(1):710. doi: 10.1038/s41419-026-08871-2 (PMC13462101; doi:10.1038/s41419-026-08871-2)
Supplement: Supplementary file 3 [file 41419_2026_8871_MOESM12_ESM.docx]

**Supplementary Table 10. List of antibody information.**

| **Reagent** | **Catalog and Source** | **Application** | **Dilution** |
| --- | --- | --- | --- |
| MTFR2 | Cat#DF14965, Affinity | IF, IHC | 1:500 |
| CD206 | Cat#14167-1-AP, Proteintech | IF, IHC | 1:1000 |
| MTFR2 | Cat#26569-1-AP, Proteintech | WB | 1:3000 |
|  |  | IF | 1:100 |
|  |  | CoIP | 5ug |
| BCAT1 | Cat#13640-1-AP, Proteintech | WB | 1:2000 |
| COXIV | Cat#11242-1-AP, Proteintech | WB | 1:10000 |
| DNM1L | Cat#12957-1-AP, Proteintech | WB | 1:6000 |
| VDAC1 | Cat#66345-1-Ig; Proteintech | WB | 1:10000 |
|  |  | IF | 1:200 |
|  |  | CoIP | 5ug |
| P65 | Cat#10745-1-AP, Proteintech | WB | 1:2000 |
|  |  | IF | 1:100 |
| P-P65 (Ser536) | Cat# AF5881, Beyotime | WB | 1:1000 |
| AKT | Cat# bsm-63324R, Bioss | WB | 1:1000 |
| P-AKT(Ser473) | Cat# LTO6000P, Litho | WB | 1:1000 |
| LAMINB1 | Cat# F0523, Selleck | WB | 1:1000 |
| TUBULIN | Cat# 66031-1-Ig, Proteintech | WB | 1:100000 |
| GAPDH | Cat# GB11002-100, Servicebio | WB | 1:3000 |
| CD68 | Cat# A24176, abclonal | FACS | 5:100 |
| CD86 | Cat# A26126, abclonal | FACS | 5:100 |
| CD206 | Cat# E-AB-F1161C, Elabscience | FACS | 5:100 |
| F4/80 | Cat# A27479, abclonal | FACS | 5:100 |
| CD206 | Cat# 12-2061-82, eBioscience | FACS | 5:100 |
| CD86 | Cat# A27138, abclonal | FACS | 5:100 |
| TLR9 | Cat# 13674, CST | IF | 1:400 |
| anti-DNA | Cat# 61014_1, PROGEN | IF | 1:200 |
