# Supplementary material for: MTFR2 promotes VDAC1 oligomerization to reprogram BCAA metabolism in tumor cells to polarize TAMs in LUAD
Source: Cell Death Dis. 2026 May 20;17(1):710. doi: 10.1038/s41419-026-08871-2 (PMC13462101; doi:10.1038/s41419-026-08871-2)
Supplement: Supplementary file 4 [file 41419_2026_8871_MOESM1_ESM.docx]

**Fig. S1. Expression profile of MTFR2 in LUAD**

**(A)** Representative IHC staining of MTFR2 in 80 paired LUAD and adjacent normal tissues. Scale bars: 500 μm (12×), 100 μm (60×).

**(B)** Bar graphs and paired-sample dot-line graphs showing H-scores of MTFR2 in 80 paired LUAD and adjacent normal tissues, with connecting lines indicating corresponding paired samples in each group (Data are represented as mean ± SD, n=80, statistical analysis by two-tailed Student’s t-test).

**(C)** Bar graphs and paired-sample dot-line graphs showing mRNA expression of MTFR2 in TCGA-LUAD paired samples, with connecting lines indicating corresponding paired samples in each group (Data are represented as mean ± SD, Normal n=58, Tumor n=478, statistical analysis by two-tailed Student’s t-test).

**(D)** Western blot of MTFR2 protein levels in B2B cells and LUAD cell lines (A549，PC9，H23，H1975，H1299), normalized to TUBULIN as an internal control (Data are represented as mean ± SD, n=3, statistical analysis by one-way ANOVA).

**(E)** qPCR analysis of MTFR2 mRNA in B2B cells and LUAD cell lines, normalized to ACTB as an internal control with B2B as the reference (Data are represented as mean ± SD, n=3, statistical analysis by one-way ANOVA).

**(F)** Kaplan-Meier survival curves showing the correlation between MTFR2 levels and OS in the LUAD TMA.

**(G)** Kaplan-Meier survival curves showing the correlation between MTFR2 levels and OS in LUAD patients based on TCGA-LUAD, GSE30219, and GSE31210 databases.

**(H)** Representative images of H&E staining and IHC staining of MTFR2 and Ki67 in subcutaneous tumor tissue sections from LLC-NC, LLC-shMTFR2, LLC-Vector, and LLC-MTFR2 groups in C57BL/6 mice. Scale bars: 2000μm (10X), 200μm (20X), 100μm (63X) (Data are represented as mean ± SD, quantification based on ≥3 random fields per group, analyzed by one-way ANOVA).

**(I)** Final tumor weights in C57BL/6 (LLC-NC, LLC-shMTFR2, LLC-Vector, and LLC-MTFR2) and NCG mice (LLC-Vector and LLC-MTFR2) (Data are represented as mean ± SD, n=5, analyzed by one-way ANOVA and two-tailed Student’s t-test).

**(J)** Flow cytometric analysis showing the percentages of CD11b^+^F4/80⁺ macrophages in LLC-NC, LLC-shMTFR2, LLC-Vector, or LLC-MTFR2 groups of C57BL/6 mice (Data are represented as mean ± SD, n=3, statistical analysis by one-way ANOVA).

**(K)** Validation of macrophage depletion efficiency by flow cytometry showing reduced CD11b^+^F4/80⁺ macrophages following clodronate administration (Data are represented as mean ± SD, n=3, analyzed by two-tailed Student’s t-test).

**(L)** Validation of macrophage depletion efficiency by qPCR showing reduced F4/80 level following clodronate administration (Data are represented as mean ± SD, n=3, analyzed by two-tailed Student’s t-test).

(ns, not significant; **P* < 0.05; ***P* < 0.01; ****P* < 0.001; *****P* < 0.0001).

**Fig. S2. Impacts of MTFR2 on tumor cell activities.**

**(A)** CCK-8 detecting effects of differential MTFR2 expression on the viability of A549, H1299, and H23 cells (Data are represented as mean ± SD, n=4, analyzed by two-way ANOVA).

**(B)** Tumor spheres of A549-Vector and A549-MTFR2 were constructed using U-shaped 96-well plates, and images were captured at 24 h, 48 h, 72 h, and 96 h, respectively. At 96 h, the spheres were incubated with Beyo3D™ 1X Calcein-AM and 1X PI dyes at 37°C for 10 min, followed by observation of fluorescence intensity and image acquisition using a laser confocal microscope. Scale bar, 500 μm (Data are represented as mean ± SD, quantification based on ≥3 random fields per group, statistical analysis by two-tailed Student’s t-test).

**(C)** Colony formation assay assessing effects of differential MTFR2 expression on the proliferation of A549 and H23 cells (Data are represented as mean ± SD, n=3, analyzed by two-tailed Student’s t-test).

(ns, not significant; **P* < 0.05; ***P* < 0.01; ****P* < 0.001; *****P* < 0.0001).

**Fig. S3. MTFR2 recruits macrophages and promotes M2 polarization in vitro.**

**(A)** Transwell assay assessing the effect of CM from H1299-NC or H1299-shMTFR2 cells on the chemotaxis of THP-1-derived macrophages.

**(B)** Transwell assay evaluating the effect of CM from H23-Vector or H23-MTFR2 cells on the chemotaxis of THP-1-derived macrophages.

**(C)** Transwell assay evaluating the effect of CM from LLC-Vector or LLC-MTFR2 cells on the chemotaxis of RAW264.7 macrophages.

**(D)** Flow cytometric analysis of the percentage of CD68⁺CD206⁺ cells in THP-1-derived macrophages after co-culture with H1299-NC or H1299-shMTFR2 cells.

**(E)** Flow cytometric analysis of the percentage of CD68⁺CD206⁺ cells in THP-1-derived macrophages after co-culture with H23-Vector or H23-MTFR2 cells.

**(F)** Flow cytometric analysis of the percentage of CD68⁺CD206⁺ cells in RAW264.7 macrophages after co-culture with LLC-Vector or LLC-MTFR2 cells.

**(G)** qPCR analysis of of M1 (CD86, iNOS) or M2 (CD163, CD206, Arg1) markers in THP-1-derived macrophages after co-culture with H1299-NC or H1299-shMTFR2 cells. Expression was normalized to ACTB as an internal control, with H1299-NC as the reference (Data are represented as mean ± SD, n=3, analyzed by two-tailed Student’s t-test).

**(H)** qPCR analysis of M1 (CD86, iNOS) or M2 (CD163, CD206, Arg1) markers in THP-1-derived macrophages after co-culture with H23-Vector or H23-MTFR2 cells. Expression was normalized to ACTB as an internal control, with H23-Vector as the reference (Data are represented as mean ± SD, n=3, analyzed by two-tailed Student’s t-test).

**(I)** qPCR analysis of M1 (CD86, iNOS) or M2 (CD163, CD206, Arg1) markers in RAW264.7 macrophages after co-culture with LLC-Vector or LLC-MTFR2 cells. Expression was normalized to ACTB as an internal control, with LLC-Vector as the reference (Data are represented as mean ± SD, n=3, analyzed by two-tailed Student’s t-test).

**(J)** Quantitative analysis of migrated cells from Figure 2B and Figure S3A-S3C (Data are represented as mean ± SD, n=3, analyzed by two-tailed Student’s t-test or one-way ANOVA).

**(K)** Quantitative analysis of the percentage of CD68⁺CD206⁺ or F4/80^+^CD206^+^ cells in macrophages from Figure 2D and Figure S3D-S3F (Data are represented as mean ± SD, n=3, analyzed by two-tailed Student’s t-test or one-way ANOVA).

(ns, not significant; **P* < 0.05; ***P* < 0.01; ****P* < 0.001; *****P* < 0.0001).

**Fig. S4. Expression Distribution and Expression Correlation Analysis of MTFR2 in the GSE131907 Single-Cell Dataset.**

**(A)** Distribution plot of MTFR2 expression in various cells from the GSE131907 single-cell dataset.

**(B-C)** Correlation analysis of MTFR2^+^ epithelial cells with immunosuppressive macrophages (spp1⁺, areg⁺) and monocytes (vcan⁺) in the GSE131907 single-cell dataset.

(ns, not significant; **P* < 0.05; ***P* < 0.01; ****P* < 0.001; *****P* < 0.0001).

**Fig. S5. MTFR2 regulates chemotaxis and M2 polarization of macrophages via BCAT1.**

**(A)** BCAT1 FPKM values detected by transcriptome sequencing in A549-Vector and A549-MTFR2 cells (Data are represented as mean ± SD, n=3, statistical analysis by two-tailed Student's t-test).

**(B)** Western blot analysis of BCAT1 protein levels in A549-Vector and A549-MTFR2 cells or A549-NC and A549-shMTFR2 cells, normalized to TUBULIN as an internal control (Data are represented as mean ± SD, n=3, statistical analysis by two-tailed Student's t-test).

**(C)** qPCR analysis of BCAT1 mRNA levels in A549-Vector and A549-MTFR2 cells or A549-NC and A549-shMTFR2 cells, normalized to ACTB as an internal control, with A549-Vector or A549-NC as the reference (Data are represented as mean ± SD, n=3, statistical analysis by two-tailed Student's t-test).

**(D)** Representative images of BCAT1 and MTFR2 IHC staining in the LUAD TMA (n=80). Scale bars: 500 μm (12×), 100 μm (60×).

**(E)** Pearson correlation between BCAT1 and MTFR2 H-scores in the LUAD TMA.

**(F)** Heatmap of differential metabolites in the supernatant of A549-Vector and A549-MTFR2 cells (n = 3).

**(G)** Flow cytometric analysis of the percentage of CD68⁺CD206⁺ cells in THP-1-derived macrophages after co-culture with A549 cells subjected to combined interventions (Vector+DMSO, Vector+BCATc Inh2 (2μM), MTFR2+DMSO, or MTFR2+BCATc Inh2).

**(H)** Transwell assay to evaluate the effect of CM from A549 cells subjected to combined interventions (Vector+DMSO, Vector+BCATc Inh2 (2μM), MTFR2+DMSO, or MTFR2+BCATc Inh2) on the chemotactic capacity of THP-1-derived macrophages.

**(I)** Quantitative analysis of the percentage of CD68⁺CD206⁺ cells in THP-1-derived macrophages from Figure 2H and Figure S5H (Data are represented as mean ± SD, n=3, analyzed by one-way ANOVA).

**(J)** Quantitative analysis of migrated cells from Figure 2I and Figure S5I (Data are represented as mean ± SD, n=3, analyzed one-way ANOVA).

**(K)** qPCR analysis of M1 (CD86, iNOS) or M2 (CD163, CD206, Arg1) markers in THP-1-derived macrophages after co-culture with A549 cells subjected to combined interventions (Vector+DMSO, Vector+BCATc Inh2 (2μM), MTFR2+DMSO, or MTFR2+BCATc Inh2). Expression was normalized to ACTB as an internal control, with A549-Vector+DMSO as the reference (Data are represented as mean ± SD, n=3, analyzed by one-way ANOVA).

(ns, not significant; **P* < 0.05; ***P* < 0.01; ****P* < 0.001; *****P* < 0.0001).

**Fig. S6. Induction of mtDNA release is a representative mitochondrial regulatory function of MTFR2**

**(A)** Mitochondrial (Mito) and cytosolic (Cyt) proteins were extracted from H1299 cells using a cell mitochondria isolation kit, with whole-cell lysates (Wh) extracted in parallel. Western blot was performed to detect the subcellular localization of MTFR2 in H1299 cells. TUBULIN and COXIV served as cytosolic and mitochondrial protein markers, respectively.

**(B)** Proteinase K protection assay to determine the localization of MTFR2 in mitochondrial membranes of H1299 cells. Highly purified mitochondria from H1299 cells were incubated with 0, 0.5, 1, 2, 5, 10, or 20 μg/ml proteinase K for 20 mins, followed by Western blot analysis of MTFR2 localization. Outer mitochondrial membrane (OMM) proteins VDAC1 and DNM1L, inner mitochondrial membrane (IMM) protein COXIV, and mitochondrial matrix (MM) protein TFAM were used as markers for OMM, IMM and MM, respectively.

**(C-D)** Super-resolution confocal microscopy to detect co-localization of MTFR2 (green) and mitochondria (magenta, Mito-Tracker) in H1299 cells. Nuclei were visualized by DAPI (blue). Scale bar, 50μm; at least 3 random fields were selected per group (C). Fluorescence intensity and co-localization of MTFR2 and mitochondria in H1299 cells were quantified using the Plot Profile plugin in ImageJ (D).

**(E)** H1299-NC and H1299-shMTFR2 cells were incubated with 200 nM Mito-Tracker at 37°C for 30 mins, and mitochondrial network structure was observed using a multimodal super-resolution live cell microscope, with representative images shown. Scale bar, 20μm; at least 3 random fields were selected per group.

**(F)** Mitochondrial average branch length, average area, and aspect ratio in H1299 cells were quantified using the Mitochondria Analyze plugin in ImageJ (Data are represented as mean ± SD, n=3, analyzed by two-tailed Student’s t-test).

**(G)** A549-Vector and A549-MTFR2 cells were incubated with 10 μM ROS probe at 37°C for 20 mins, and ROS fluorescence intensity was detected via the FITC channel using flow cytometry.

**(H)** A549-Vector and A549-MTFR2 cells were incubated with 100 nM TMRM probe at 37°C for 30 mins, and TMRM fluorescence intensity was detected via the PE channel using flow cytometry.

**(I)** Quantitative analysis of relative positive rates from flow cytometric analysis of ROS (G) and TMRM (H) in A549-Vector and A549-MTFR2 cells, normalized to A549-Vector (data presented as mean±SD, n=3, analyzed by two-tailed Student’s t-test).

**(J)** A549-Vector and A549-MTFR2 cells were incubated with 10 μM ROS probe (green) and 200 nM Mito-Tracker (magenta) at 37°C for 30 mins, followed by observation of ROS fluorescence intensity using super-resolution confocal microscopy, with representative images shown. Nuclei were counterstained with Hoechst (blue). Scale bar, 50μm; at least 3 random fields were selected per group.

**(K)** A549-Vector and A549-MTFR2 cells were incubated with 100 nM TMRM probe (red) at 37°C for 30 mins, followed by observation of TMRM fluorescence intensity using super-resolution confocal microscopy, with representative images shown. Nuclei were counterstained with Hoechst (blue). Scale bar, 50μm; at least 3 random fields were selected per group.

**(L)** Quantitative analysis of relative fluorescence intensities of ROS (Fig. S6 J) and TMRM (Fig. S6 K) in A549-Vector and A549-MTFR2 cells, normalized to A549-Vector (data presented as mean±SD, n=3, analyzed by two-tailed Student’s t-test).

(ns, not significant; **P* < 0.05; ***P* < 0.01; ****P* < 0.001; *****P* < 0.0001).

**Fig. S7. MTFR2 Engages the Leu125 Residue of VDAC1.**

**(A)** Coomassie brilliant blue-stained gel: A549 cell proteins were extracted, and MTFR2-binding proteins were identified using immunoprecipitation combined with IP-LC-MS/MS analysis.

**(B-C)** Confocal microscopy to detect co-localization of MTFR2 (red) and VDAC1 (green) in H1299-NC and H1299-shMTFR2 cells. Nuclei were visualized by DAPI (blue). Scale bar, 20μm; at least 3 random fields were selected per group (B). Pearson's correlation coefficients for MTFR2 and VDAC1 co-localization in H1299-NC and H1299-shMTFR2 cells were determined using the Coloc 2 plugin in ImageJ (C).

**(D)** PyMOL visualization of AlphaFold3-predicted interaction sites between MTFR2 and VDAC1.

**(E)** A549 cells were co-transfected with Myc-MTFR2 and Flag-VDAC1-WT, β8-SCF fragment, or L125A mutant, respectively, followed by PLA to detect MTFR2-VDAC1 interaction (red). Nuclei were counterstained with DAPI (blue). Scale bars, 30 μm. Fluorescence intensity was quantified using ImageJ (at least 3 random fields per group; Data are represented as mean ± SD, n=3, analyzed by two-tailed Student's t-test).

**(F-G)** Pearson correlation analysis of MTFR2-VDAC1 and BCAT1-VDAC1 expression correlations in TCGA-LUAD.

**(H)** mtDNA signals outside mitochondrial boundaries and excluding nuclear regions of Figure 5E-5G were quantified using ImageJ (Data are represented as mean ± SD, n=3, analyzed by two-tailed Student’s t-test).

**(I)** qPCR analysis of M1 (CD86, iNOS) or M2 (CD163, CD206, Arg1) markers in THP-1-derived macrophages after co-culture with A549-MTFR2 cells subjected to combined interventions (VDAC1-WT, VDAC1-L125A, DMSO, or VBIT-4 10μM). Expression was normalized to ACTB as an internal control, with VDAC1-WT or DMSO as the reference (Data are represented as mean ± SD, n = 3, analyzed by two-tailed Student’s t-test).

**(J-K)** Transwell assay evaluating the effect of CM from A549-MTFR2 cells subjected to different interventions (VDAC1-WT and VDAC1-L125A, or VBIT4 (10μM) and DMSO) on the chemotaxis of THP-1-derived macrophages (Data are represented as mean ± SD, n=3, analyzed by two-tailed Student’s t-test).

(ns, not significant; **P* < 0.05; ***P* < 0.01; ****P* < 0.001; *****P* < 0.0001).

**Fig. S8. Cytoplasmic mtDNA drives macrophage M2 polarization and chemotaxis by upregulating BCAT1 via the TLR9-NF-κB pathway.**

**(A)** Bubble plot of Reactome functional enrichment analysis for differentially expressed gene sets between A549-Vector and A549-MTFR2 cells: The c2.cp.reactome.v7.4.symbols.gmt subset was downloaded from the Molecular Signatures Database (DOI:10.1093/bioinformatics/btr260, <http://www.gsea-msigdb.org/gsea/downloads.jsp>). Enrichment analysis was performed using the R package clusterProfiler (version 3.14.3) with parameters set as follows: minimum gene set size = 5, maximum gene set size = 5000, |Log₂FC| > 1 and *P* < 0.05. n=3.

**(B)** GSEA analysis based on WIKI Pathway: GSEA software (version 3.0) was obtained from the GSEA website (DOI:10.1073/pnas.0506580102, <http://software.broadinstitute.org/gsea/index.jsp>). The c2.cp.wikipathways.v7.4.symbols.gmt subset was downloaded from the Molecular Signatures Database (DOI:10.1093/bioinformatics/btr260, <http://www.gsea-msigdb.org/gsea/downloads.jsp>). Based on the sequencing gene set expression profiles of A549-Vector and A549-MTFR2 cells, the parameters were set as follows: minimum gene set size = 5, maximum gene set size = 5000, 1000 permutations, and *P* value < 0.1. n=3.

**(C-E)** Confocal fluorescence microscopy to detect co-localization of cGAS (red) and mtDNA (green) in A549-MTFR2 cells. Nuclei were visualized by DAPI (blue). Scale bar, 50μm; at least 3 random fields were selected per group (C). Pearson's correlation coefficients for cGAS and mtDNA co-localization were determined using the Coloc 2 plugin in ImageJ (D). Fluorescence intensity profiles along the indicated lines were generated using the Plot Profile plugin (E).

**(F-H)** Confocal fluorescence microscopy to detect co-localization of NLRP3 (red) and mtDNA (green) in A549-MTFR2 cells. Nuclei were visualized by DAPI (blue). Scale bar, 50μm; at least 3 random fields were selected per group (F). Pearson's correlation coefficients for NLRP3 and mtDNA co-localization were determined using the Coloc 2 plugin in ImageJ (G). Fluorescence intensity profiles along the indicated lines were generated using the Plot Profile plugin (H).

**(I)** GSEA analysis based on HALLMARK: The h.all.v7.4.symbols.gmt subset was downloaded from the Molecular Signatures Database (DOI:10.1093/bioinformatics/btr260, <http://www.gsea-msigdb.org/gsea/downloads.jsp>). Based on the sequencing gene set expression profiles of A549-Vector and A549-MTFR2 cells, the parameters were set as follows: minimum gene set size = 5, maximum gene set size = 5000, 1000 permutations, and *P* < 0.1. n=3.

**(J-L)** A549-MTFR2 cells pretreated with VBIT4 (10μM), DNase I, or vehicle were analyzed for P65 (red) localization using Confocal fluorescence microscopy. Nuclei were visualized by DAPI (blue). Scale bar, 50μm; at least 3 random fields were selected per group (J). Fluorescence intensity profiles along the indicated lines were generated using the Plot Profile plugin (K). Pearson's correlation coefficients for P65 and DAPI co-localization were determined using the Coloc 2 plugin in ImageJ (L).

**(M)** qPCR analysis of BCAT1 in A549-MTFR2-Vehicle, A549-MTFR2-VBIT4, and A549-MTFR2-DNaseI cells. Expression was normalized to ACTB as an internal control, with A549-MTFR2-Vehicle as the reference (Data are represented as mean ± SD, n=3, analyzed by one-way ANOVA).

**(N)** Kaplan-Meier survival curves showing the correlation between VDAC1 or TLR9 levels and OS in LUAD patients based on TCGA-LUAD.

(ns, not significant; **P* < 0.05; ***P* < 0.01; ****P* < 0.001; *****P* < 0.0001).

**Fig. S9. Secondary mass spectrometry (MS/MS) spectra of major metabolites in the BCAA metabolic pathway in the supernatant of A549-Vector and A549-MTFR2 cells.**

**Fig. S10. Dose-dependent induction of M2 macrophage polarization by KIC and KMV and its reversal by AKT inhibition.**

**(A-B)** THP-1-derived M0 macrophages were treated with increasing concentrations of KIC or KMV (0, 25, 50, 100, and 200 μM) for 24 h, and the percentage of CD68⁺CD206⁺ M2 macrophages was determined by flow cytometry.

**(C-E)** M2 macrophages or M0 macrophages treated with exogenous KIC or KMV (100 μM) were subjected to gradient concentrations of the AKT inhibitor MK-2206 (0, 1, 5, and 10 μM), followed by flow cytometric analysis of CD68⁺CD206⁺ cell proportions (Data are represented as mean ± SD, n=3, analyzed by one-way ANOVA).

(ns, not significant; **P* < 0.05; ***P* < 0.01; ****P* < 0.001; *****P* < 0.0001).

**Fig. S11. Targeting BCAT1 or depleting macrophages inhibits M2 polarization and tumor-promoting effects mediated by KIC and KMV.**

**(A-D)** M2 macrophages **(A)** or M0 macrophages treated with exogenous KIC **(B)** or KMV **(C)** (100 μM) were exposed to gradient concentrations of the AKT inhibitor MK-2206 (0, 1, 5, and 10 μM) and subsequently co-cultured with A549 tumor spheroids. At 96 h, spheroids were stained with Beyo3D™ 1× Calcein-AM and 1× PI at 37°C for 10 min and imaged by laser confocal microscopy. Scale bars, 50 μm; at least 3 random fields were selected per group. Quantitative Analysis of PI/Calcein Fluorescence Intensity Ratio in Fig. S11A-S11C **(D)** (Data are represented as mean ± SD, n=3, analyzed by one-way ANOVA).

**(E)** Flow cytometric analysis of tumor-associated macrophages in subcutaneous tumors from C57BL/6 mice showing the percentage of CD11b⁺F4/80⁺ cells in LLC-Vector and LLC-BCAT1 groups (Left) and in LLC-MTFR2-bearing mice treated with vehicle, BCATc Inh2 (30 mg/kg), or BCATc Inh2 plus clodronate liposomes (1 mg/200 μL) (Right).

**(F)** Flow cytometric analysis of M2 macrophage infiltration in subcutaneous tumors from C57BL/6 mice. Percentage of CD11b⁺F4/80⁺CD86⁺ cells in LLC-Vector and LLC-BCAT1 groups (Left). Percentage of CD11b⁺F4/80⁺CD86⁺ macrophages in LLC-MTFR2-bearing mice treated with vehicle, BCATc Inh2 (30 mg/kg), or BCATc Inh2 plus clodronate liposomes (1 mg/200 μL) (Right).

**(G)** Quantification of Flow cytometric analysis in Fig. 8G and Fig. S11E-F (Data are represented as mean ± SD, n=3, analyzed by one-way ANOVA).

(ns, not significant; **P* < 0.05; ***P* < 0.01; ****P* < 0.001; *****P* < 0.0001).

**Fig. S12. Flow cytometry gating strategy.**

**(A)** FACS plots illustrate the gating strategy beginning with exclusion of debris and doublets and pre-selection of target populations based on light scatter characteristics, followed by selection of 7AAD-negative live cells within this gate, identification of CD11b⁺F4/80⁺ macrophages, and rectangular gating to distinguish CD86⁺ (M1) and CD206⁺ (M2) subsets.

**Fig. S13. Overexpression or knockdown efficiency.**

**(A-B)** A549 or H1299 cells were transfected with negative control (NC) siRNA or siRNA targeting MTFR2. After 48 hours, total proteins were extracted, and Western blot was performed to detect the knockdown efficiency of siRNA, with TUBULIN as an internal control for normalization.

**(C-E)** A549 and H1299 cells stably infected with shMTFR2 or NC lentivirus were constructed. Western blot was used to detect the knockdown efficiency of MTFR2 protein, with TUBULIN as an internal control for normalization.

**(F-H)** A549 or H1299 cells stably infected with MTFR2 or Vector lentivirus were constructed. Western blot was performed to detect the overexpression efficiency of MTFR2 protein, with TUBULIN as an internal control for normalization.

**(I)** Quantitative analysis of grayscale values from Figure S2A-H (Data are represented as mean ± SD, n=3, analyzed by two-tailed Student’s t-test or one-way ANOVA).

**(J)** A549 cells were transfected with negative control (NC) siRNA or siRNA targeting BCAT1. After 48 hours, total proteins were extracted, and Western blot was performed to detect the knockdown efficiency of siRNA, with TUBULIN as an internal control for normalization (Data are represented as mean ± SD, n=3, analyzed by two-tailed Student’s t-test).

**(K)** A549 or H1299 cells transfected with VDAC1 or Vector plasmid were constructed. Western blot was performed to detect the overexpression efficiency of VDAC1 protein, with TUBULIN as an internal control for normalization (Data are represented as mean ± SD, n=3, analyzed by one-way ANOVA).

**Fig. S14. Raw bright-field images of Western Blot.**

**Supplementary Table 1.** **Information on MTFR2 expression and clinicopathological characteristics in 80 patients with LUAD.**

**Supplementary Table 2.** **Univariate and multivariate COX analysis of Overall survival in 80 patients with LUAD.**

**Supplementary Table 3. Information on MTFR2/CD206 co-expression and clinicopathological characteristics in 80 patients with LUAD.**

**Supplementary Table 4. Figure 4A MTFR2 interacting proteins and MitoCarta3.0 Database.**

**Supplementary Table 5.** **Figure 6C TLR9 associated pathway and BCAT1 associated TF.**

**Supplementary Table 6. Patient information of organoids construction.**

**Supplementary Table 7. Patient information of Tissue microarray.**

**Supplementary Table 8. List of siRNA and shRNA sequences.**

**Supplementary Table 9.** **List of primer sequences for qPCR.**

**Supplementary Table 10. List of** **antibody information.**

**Supplementary Table 11. Differential metabolite list.**
