# Supplementary material for: MTFR2 promotes VDAC1 oligomerization to reprogram BCAA metabolism in tumor cells to polarize TAMs in LUAD
Source: Cell Death Dis. 2026 May 20;17(1):710. doi: 10.1038/s41419-026-08871-2 (PMC13462101; doi:10.1038/s41419-026-08871-2)
Supplement: Supplementary file 6 [file 41419_2026_8871_MOESM3_ESM.docx]

**Supplementary Table 1. Information on MTFR2 expression and clinicopathological characteristics in 80 patients with LUAD.**

| Characteristics | MTFR2^Low^ (n=40) | MTFR2^High^ (n=40) | *P* value |
| --- | --- | --- | --- |
| Gender, n (%) |  |  | 0.263 |
| female | 17 (21.2%) | 22 (27.5%) |  |
| male | 23 (28.7%) | 18 (22.5%) |  |
| Age, mean ± sd | 60.225 ± 9.5097 | 57.95 ± 8.9212 | 0.273 |
| T, n (%) |  |  | 0.873 |
| T1 | 10 (12.5%) | 13 (16.2%) |  |
| T2 | 20 (25%) | 19 (23.8%) |  |
| T3 | 8 (10%) | 6 (7.5%) |  |
| T4 | 2 (2.5%) | 2 (2.5%) |  |
| N, n (%) |  |  | 0.341 |
| N0 | 18 (22.5%) | 22 (27.5%) |  |
| N1 | 7 (8.8%) | 9 (11.2%) |  |
| N2 | 15 (18.8%) | 9 (11.2%) |  |
| M, n (%) |  |  | **0.014** |
| M0 | 36 (45%) | 27 (33.8%) |  |
| M1 | 4 (5%) | 13 (16.2%) |  |
| Stage, n (%) |  |  | **0.044** |
| I | 10 (12.5%) | 9 (11.2%) |  |
| II | 9 (11.2%) | 10 (12.5%) |  |
| III | 17 (21.2%) | 8 (10%) |  |
| IV | 4 (5%) | 13 (16.2%) |  |
| Location, n (%) |  |  | 0.256 |
| right | 21 (26.2%) | 26 (32.5%) |  |
| left | 19 (23.8%) | 14 (17.5%) |  |
| MTFR2, median (IQR) | 15.369 (4.9511, 30.952) | 66.142 (52.951, 87.797) | **< 0.001** |
