# Supplementary material for: MTFR2 promotes VDAC1 oligomerization to reprogram BCAA metabolism in tumor cells to polarize TAMs in LUAD
Source: Cell Death Dis. 2026 May 20;17(1):710. doi: 10.1038/s41419-026-08871-2 (PMC13462101; doi:10.1038/s41419-026-08871-2)
Supplement: Supplementary file 7 [file 41419_2026_8871_MOESM4_ESM.docx]

#### Supplementary Table 2. Univariate and multivariate COX analysis of Overall survival in 80 patients with LUAD.

| Characteristics | Total (n) | Univariate analysis | |  | Multivariate analysis | |
| --- | --- | --- | --- | --- | --- | --- |
|  |  | Hazard ratio (95% CI) | *P* value |  | Hazard ratio (95% CI) | *P* value |
| Gender | 80 |  |  |  |  |  |
| male | 41 | Reference |  |  | Reference |  |
| female | 39 | 0.782 (0.448 - 1.364) | 0.386 |  | 0.596 (0.291 - 1.220) | 0.157 |
| Age | 80 | 1.014 (0.985 - 1.045) | 0.336 |  | 1.065 (1.024 - 1.107) | 0.002 |
| T | 80 |  |  |  |  |  |
| T1 | 23 | Reference |  |  | Reference |  |
| T2 | 39 | 1.092 (0.575 - 2.073) | 0.788 |  | 1.141 (0.564 - 2.308) | 0.714 |
| T3 | 14 | 1.316 (0.575 - 3.012) | 0.516 |  | 1.870 (0.704 - 4.967) | 0.209 |
| T4 | 4 | 0.334 (0.044 - 2.532) | 0.289 |  | 0.252 (0.026 - 2.408) | 0.232 |
| N | 80 |  |  |  |  |  |
| N0 | 40 | Reference |  |  | Reference |  |
| N1 | 16 | 2.523 (1.216 - 5.235) | **0.013** |  | 3.400 (1.270 - 9.101) | **0.015** |
| N2 | 24 | 3.697 (1.921 - 7.115) | **< 0.001** |  | 6.125 (1.962 - 19.125) | **0.002** |
| M | 80 |  |  |  |  |  |
| M0 | 66 | Reference |  |  | Reference |  |
| M1 | 14 | 3.712 (1.939 - 7.109) | **< 0.001** |  | 0.534 (0.103 - 2.762) | 0.454 |
| Stage | 80 |  |  |  |  |  |
| IV | 17 | Reference |  |  | Reference |  |
| I | 19 | 0.111 (0.042 - 0.291) | **< 0.001** |  | 0.087 (0.012 - 0.635) | **0.016** |
| II | 19 | 0.244 (0.111 - 0.534) | **< 0.001** |  | 0.072 (0.013 - 0.402) | **0.003** |
| III | 25 | 0.407 (0.202 - 0.819) | **0.012** |  | 0.127 (0.018 - 0.898) | **0.039** |
| Location | 80 |  |  |  |  |  |
| right | 47 | Reference |  |  | Reference |  |
| left | 33 | 1.140 (0.652 - 1.995) | 0.645 |  | 1.647 (0.851 - 3.189) | 0.138 |
| MTFR2 | 80 |  |  |  |  |  |
| Low | 20 | Reference |  |  | Reference |  |
| Stable | 40 | 1.175 (0.558 - 2.474) | 0.671 |  | 1.210 (0.511 - 2.866) | 0.664 |
| High | 20 | 2.542 (1.158 - 5.584) | **0.020** |  | 3.187 (1.265 - 8.026) | **0.014** |
