# Supplementary material for: MTFR2 promotes VDAC1 oligomerization to reprogram BCAA metabolism in tumor cells to polarize TAMs in LUAD
Source: Cell Death Dis. 2026 May 20;17(1):710. doi: 10.1038/s41419-026-08871-2 (PMC13462101; doi:10.1038/s41419-026-08871-2)
Supplement: Supplementary file 8 [file 41419_2026_8871_MOESM5_ESM.docx]

**Supplementary Table 3. Information on MTFR2/CD206 co-expression and clinicopathological characteristics in 80 patients with LUAD.**

| Characteristics | MTFR2^Low^CD206^Low^ (n=23) | MTFR2^High^CD206^High^ (n=23) | *P* value |
| --- | --- | --- | --- |
| Gender, n (%) |  |  | 0.238 |
| female | 9 (19.6%) | 13 (28.3%) |  |
| male | 14 (30.4%) | 10 (21.7%) |  |
| Age, mean ± sd | 57.696 ± 9.9792 | 58.435 ± 10.153 | 0.805 |
| T, n (%) |  |  | 0.989 |
| T1 | 6 (13%) | 7 (15.2%) |  |
| T2 | 11 (23.9%) | 10 (21.7%) |  |
| T3 | 5 (10.9%) | 5 (10.9%) |  |
| T4 | 1 (2.2%) | 1 (2.2%) |  |
| N, n (%) |  |  | 0.503 |
| N0 | 12 (26.1%) | 13 (28.3%) |  |
| N1 | 5 (10.9%) | 7 (15.2%) |  |
| N2 | 6 (13%) | 3 (6.5%) |  |
| M, n (%) |  |  | **0.032** |
| M0 | 21 (45.7%) | 15 (32.6%) |  |
| M1 | 2 (4.3%) | 8 (17.4%) |  |
| Stage, n (%) |  |  | 0.066 |
| I | 5 (10.9%) | 5 (10.9%) |  |
| II | 8 (17.4%) | 8 (17.4%) |  |
| III | 8 (17.4%) | 2 (4.3%) |  |
| IV | 2 (4.3%) | 8 (17.4%) |  |
| Location, n (%) |  |  | 0.234 |
| right | 11 (23.9%) | 15 (32.6%) |  |
| left | 12 (26.1%) | 8 (17.4%) |  |
| OS, n (%) |  |  | **0.016** |
| alive | 13 (28.3%) | 5 (10.9%) |  |
| dead | 10 (21.7%) | 18 (39.1%) |  |
| MTFR2, median (IQR) | 18.052 (8.3706, 29.628) | 63.804 (53.646, 96.059) | **< 0.001** |
| CD206, median (IQR) | 0.5439 (0.19915, 0.7346) | 3.7159 (2.0949, 12.462) | **< 0.001** |
